# Transcranial electrical stimulation (TES) in human motor Optimization: Mechanisms, safety, and emerging applications

**DOI:** 10.1016/j.bbrep.2025.102055

**Published:** 2025-06-02

**Authors:** Jingfeng Wang, Li Wu, Mingming Sun, Yuxiang Wu

**Affiliations:** a Institute of Intelligent Sport and Proactive Health, Department of Health and Physical Education, Jianghan University, Wuhan, 430056, China; b Beijing Institute of Nanoenergy and Nanosystems, Chinese Academy of Sciences, Beijing, 101400, China

**Keywords:** TES, Neuromodulation, Sports performance, Safety

## Abstract

Non-invasive brain stimulation (NIBS) has emerged as a rapidly advancing field, offering promising therapeutic interventions for a range of neurological disorders while effectively bridging the gap between laboratory research and clinical applications. Among NIBS technologies, transcranial electrical stimulation (TES) stands out as a notable example, utilizing electrodes of varying sizes to deliver low-intensity electrical currents to specific regions of the cerebral cortex. This technique facilitates the modulation of neuronal excitability, regulation of brainwave activity, promotion of neural remodeling and repair, enhancement of cerebral blood flow, and improvement of brain-muscle connectivity. Despite its potential, current research on the effects of TES on motor function across diverse populations, particularly from a central nervous system perspective, remains limited. This review seeks to establish a theoretical framework for the future advancement of TES technology in sports science, elucidate the neurophysiological mechanisms underlying various TES modalities, and synthesize the most recent experimental findings from the past two decades regarding its impact on physical fitness, motor skill acquisition, and recovery in different populations.

## Highlights

• Develop an EEG framework for exercise science to advance precise nerve regulation tech.
• Study cross-scale EEG stimulation effects on brain waves and neural remodeling.
• Summarize 20 years of evidence on brain stimulation's role in motor learning and recovery.
• Explain how brain stimulation boosts blood flow, brain-muscle sync, and performance.

## 1 Introduction

**Fig. 1:**
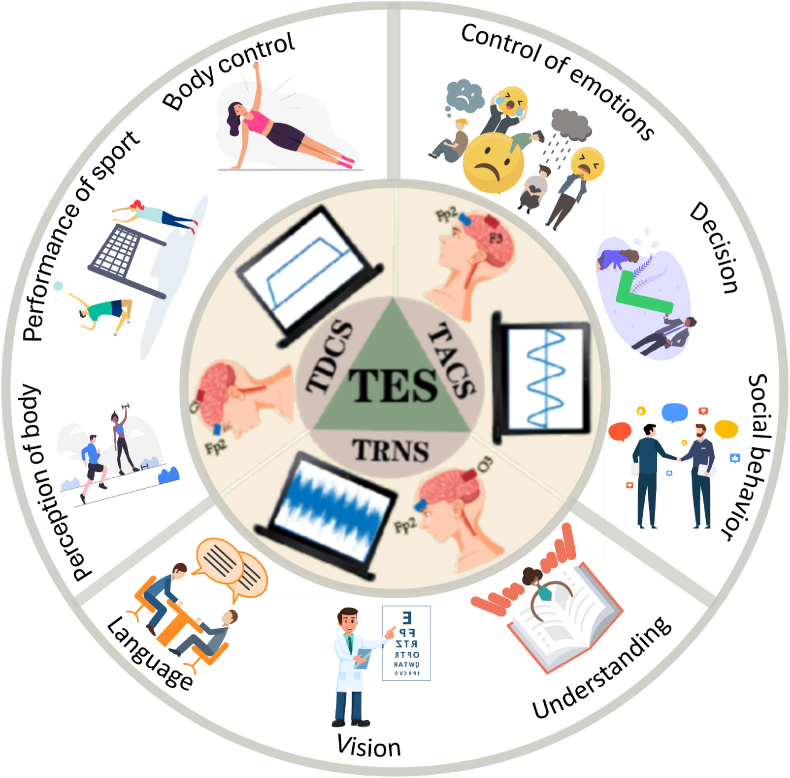
Electrode placement, current waveform and influencing factors for different TES types.

As sporting events continue to expand globally, athletes and sports teams face growing challenges in enhancing performance and achieving competitive results in a timely, safe, and efficient manner. Traditional training methods, which primarily focus on strengthening the cardiovascular system, lungs, and muscles, have limitations in addressing the complex demands of modern sports. In this context, advancements in neuromodulation technology, particularly transcranial electrical stimulation (TES), offer a promising alternative. TES employs electrodes of varying sizes to deliver low-intensity electrical currents to specific brain regions, modulating cortical excitability, enhancing brain-muscle communication, and improving the central nervous system's ability to regulate physiological functions. Fig. 1.

Transcranial electrical stimulation (TES) encompasses three primary modalities: transcranial random noise stimulation (tRNS), transcranial alternating current stimulation (tACS), and transcranial direct current stimulation (tDCS). Among these, tDCS modulates neuronal activity by inducing either hyperpolarization or depolarization of the resting membrane potential, depending on the stimulus polarity. Additionally, tDCS can alter neuronal excitability in specific brain regions and promote synaptic plasticity, offering potential applications in both research and clinical settings. [1]. Transcranial alternating current stimulation (tACS) modulates neural activity by entraining large populations of neurons and regulating neural oscillations through biphasic, sinusoidal currents. A variant of tACS, transcranial random noise stimulation (tRNS), leverages stochastic resonance with “white noise” properties to influence brain oscillatory rhythms. Current evidence indicates that TES administration may lead to transient adverse effects, such as localized itching, mild erythema, warmth, or paresthesia on the scalp. However, these symptoms are temporary, typically subsiding after stimulation ceases, and no severe side effects or irreversible brain damage have been reported [2].

Despite the growing interest in transcranial electrical stimulation (TES), research examining its impact on motor performance across diverse populations from a central nervous system perspective remains limited. To address this gap, this paper aims to establish a scientific foundation and provide guidelines for the clinical application, promotion, and enhancement of sports performance through TES. First, the neurophysiological mechanisms underlying TES effects on the cerebral cortex are elucidated. Next, the most recent scientific advancements over the past two decades in the domains of physical fitness enhancement, motor skill acquisition, and motor recovery are summarized and analyzed. Finally, the current applications of TES in sports performance and its safety profile are critically examined. The overarching goal of this study is to provide a robust scientific basis and practical recommendations for the clinical utilization, development, and optimization of TES technology.

## 2 Transcranial direct current stimulation

Which non-invasive techniques in contemporary neuroscience can effectively and safely enhance motor function? Transcranial electrical stimulation (TES) is a non-invasive brain stimulation method that modulates neuronal activity. The three primary forms of TES are transcranial direct current stimulation (tDCS), transcranial alternating current stimulation (tACS), and transcranial random noise stimulation (tRNS). Among these, tDCS involves the application of low-intensity direct current (DC) through electrodes placed on the scalp, altering neuronal excitability. tDCS has been widely utilized in research on neurological and psychiatric disorders, including depression, schizophrenia, obsessive-compulsive disorder, epilepsy, Alzheimer's disease, Parkinson's disease, stroke, substance addiction, and attention disorders. Additionally, it has applications in cognitive function, autonomic nervous system regulation, appetite control, energy expenditure, motor performance, and motor learning. tDCS is functional, repeatable, and supported by portable, user-friendly equipment. While it may cause transient side effects such as localized itching, erythema, and warmth on the scalp, these symptoms are mild, reversible, and typically subside after stimulation ceases, with no evidence of severe adverse effects or irreversible brain damage.

The use of electrical stimulation in medical research dates back to antiquity, with historical evidence suggesting that Roman physicians employed the mild electrical discharges from electric rays to treat conditions such as headaches and gout. [3]. In the 18th century, John Wesley pioneered the therapeutic application of electroconvulsive therapy for pain management in clinical settings. Building upon this foundation, Luigi Galvani conducted seminal experiments demonstrating the physiological effects of electrical impulses through the induction of muscular contractions in amphibian specimens, specifically in isolated frog legs. [4]. The systematic application of transcranial electrical stimulation (TES) in both animal and human studies commenced in the 19th and 20th centuries. In the mid-20th century, Merton and Morton pioneered the concept of TES, utilizing this technique to stimulate the human motor cortex. Their work suggested that the underlying mechanism involves the application of a single, non-sustained, high-intensity pulse to modulate the motor cortex, thereby enhancing motor function. This approach focuses on cortical modulation through brief, high-intensity pulses, leading to measurable improvements in motor performance [5].

**Fig. 2:**
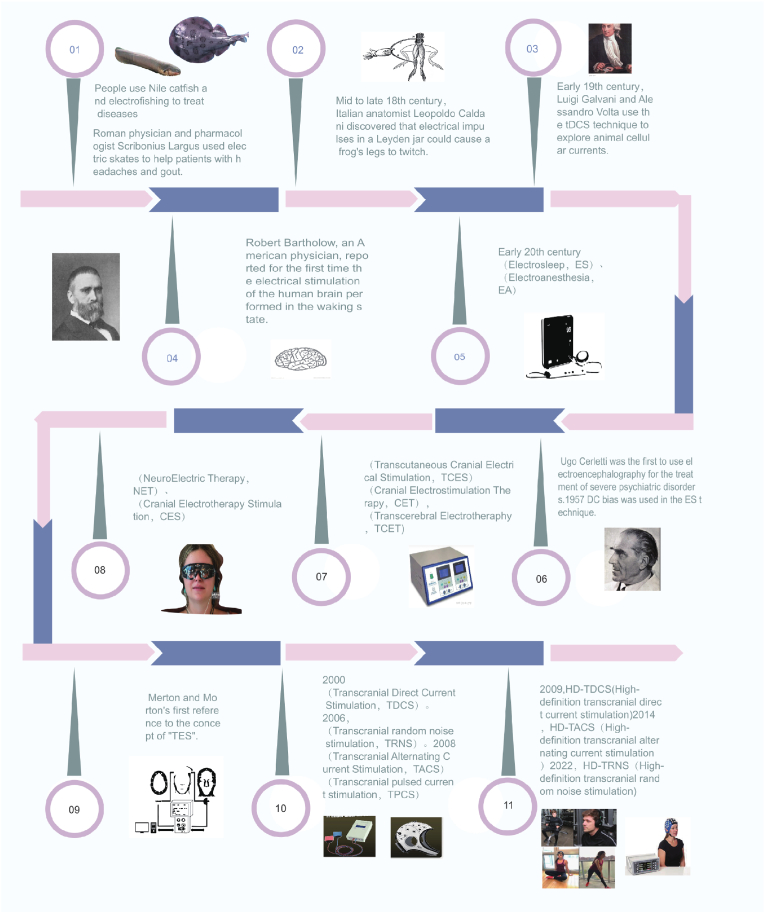
The development history of TES [7,8,9,10,11,12,13].

TES has evolved historically and innovatively from “electro-sleep,” “electro-anaesthesia,” and “electro-convulsive therapy” procedures [6] to become a highly valued technology in the last century, as shown in Fig. 2.

### 2.1 Parameters of transcranial direct current stimulation

Stimulation parameters to be determined when undergoing tDCS treatment include electrode lead configuration (involving the skin contact area/size and location of all electrodes), stimulation intensity, and duration.

tDCS modulates cortical excitability and remodels neuronal activity by applying low-intensity DC currents (0.5–2 mA) to specific brain areas [14] through two or more electrodes placed on the scalp. Fig. 4a. A single tDCS stimulation session lasts about 10–30 min, excluding the 10–30 s of instability at the start and end of the administered stimulus; The total charge is 15–100 μC/cm^2^;Recently, Nitsche et al. employed 4 mA TDC for the clinical treatment of patients with ischemic stroke in an effort to investigate the safety and tolerability of higher-intensity tDCS applied to the human body. They were successful in their intervention efforts [15]. Regarding the post-induction effect of tDCS, it has been shown that the post-stimulation effect of 15 min of tDCS can last for 30 min [16] or 90 min^154^.

Recently, high-definition tDCS (High definition-transcranial direct current stimulation, HD-tDCS) [17] Fig. 4b and individualized HD-tDCS [18] (individualized High definition-transcranial direct current stimulation, individualized HD-tDCS) Fig. 4c have been proposed as centralised forms of tDCS. HD-tDCS employs an array of five 1 × 1 cm ring electrodes to deliver focal current to targeted brain regions. In this configuration, the central electrode serves as the active stimulation site, while the surrounding four electrodes function as return pathways. Compared to conventional tDCS, HD-tDCS and its individualized variants enable more precise spatial targeting of cortical regions while minimizing scalp contact area. This enhanced focality translates to measurable neurophysiological changes and therapeutic benefits across both clinical and non-clinical populations. Enhancing the effect of tDCS stimulation can be achieved by increasing the stimulation intensity, duration, or by repeating the stimulation protocol [19]. Recent studies have re-examined the “ceiling effect” — the theoretical limit of therapeutic efficacy achievable through singular high-intensity or prolonged stimulation — demonstrating its limitations in transcranial electrical stimulation (TES) [20]. Current tDCS parameter optimization remains preliminary, with critical variables such as stimulation mode, electrode configuration (size, placement), and anatomical variations (skull thickness, cerebrospinal fluid volume) collectively modulating intracranial electric field distributions. These interdependent factors amplify field heterogeneity, thereby driving divergent experimental and clinical outcomes [21].

### 2.2 Physiological mechanisms of transcranial direct current stimulation

**Fig. 3:**
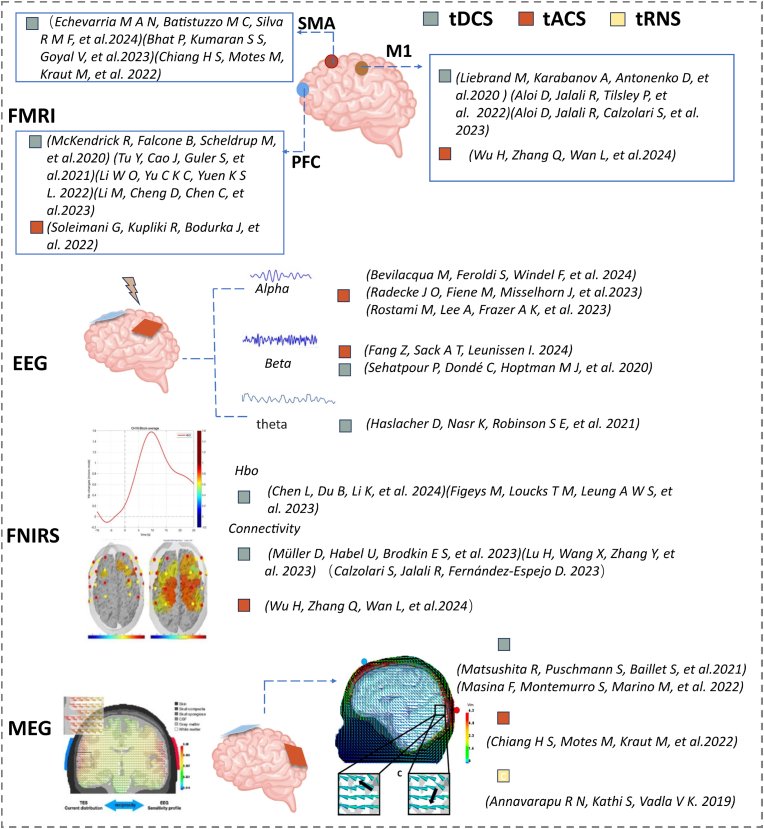
Summary of applications of TES electrode placement in different brain regions based on Functional Magnetic Resonance Imaging (fMRI) [28,29,30,31,32,33,34,35,36], Electroencephalogram (EEG) [37,38,39,40,41,42], Functional Near-Infrared Spectroscopy (fNIRS) [43,44,45,46,47,48]and Magnetoencephalography (MEG) imaging techniques [49,44,50,51].

The integration of neuromodulation with neurophysiological and brain imaging techniques offers a wealth of opportunities for investigating the physiological underpinnings of brain modulation [22,23,24,25,26,27]. This is crucial for delving deeply into the intricate network mechanisms underlying functional brain disorders. The effectiveness of tDCS on cerebral hemodynamics can be predicted using a range of acquired neurophysiological, brain imaging, and clinical markers. Fig. 3.

**Fig. 4:**
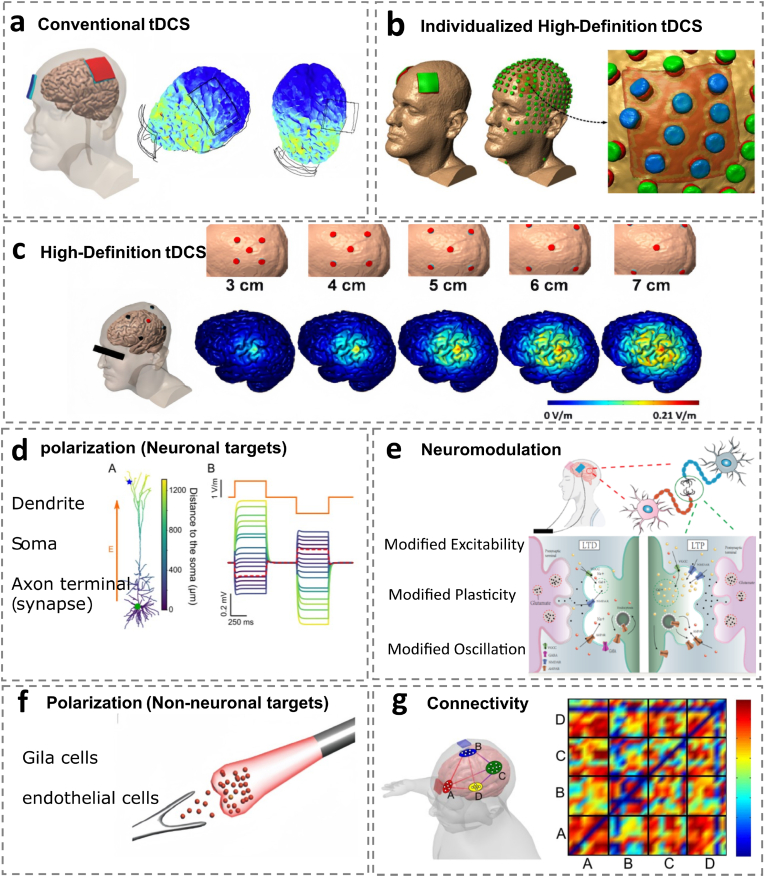
Schematic representation of the different types of tDCS and their physiological mechanisms.(a)Conventional tDCS [52].(b)Individualized High-Definition tDCS [47].(c)High-Definition tDCS [53].(d)Differential polarization of cortical pyramidalneuron dendrites through weak extracellular fields [54].(e)The effects of tDCS on individual neurons are neurochemically modulated to include LTP and LTD [55].(f)Astrocytes as a target of tDCS to treat depression [56,57,58,52]. (g)tDCS-induced changes in brain synchronization and topological functional organization [52,59].

TDCS increases neural network oscillations, motor cortical excitability [60]、neuroplasticity, and the resting membrane potential of subthreshold neurons. Fig. 4e [61].

TDCS is classified based on polarity into anodal (a-tDCS) and cathodal (c-tDCS) stimulation. Single-neuron recording studies demonstrate that a-tDCS applied to the motor cortex induces depolarization of resting membrane potentials, thereby increasing cortical excitability. Conversely, c-tDCS application to the same region triggers hyperpolarization of resting membrane potentials, thereby decreasing cortical excitability. [62]. It is worth noting that this regulation is not limited to the stimulation target area, but can also affect distant brain areas through intercortical connections (such as the transcallosal inhibitory effect of stimulation of the contralateral hemisphere of the premotor cortex) [63]. When a-tDCS is applied, glutamate undergoes depolarization and its concentration increases. This activates both *N*-methyl-d-aspartate receptors (NMDARs) and voltage-gated calcium channels (VGCCs), thereby elevating intracellular Ca^2+^ concentrations and inducing long-term potentiation (LTP). Notably, while sustained, homogeneous Ca^2+^ elevation promotes long-term depression (LTD), transient but pronounced Ca^2+^ spikes preferentially trigger LTP. Conversely, cathodal tDCS (c-tDCS) induces glutamate hyperpolarization and reduces its concentration. This activates GABA receptors, inhibits NMDAR and VGCC activity, and diminishes Ca^2+^ influx, ultimately leading to LTD [64].

The effects of tDCS on neuronal activity can be observed across multiple structural levels, including dendrites, cell bodies (soma), and axon terminals. Fig. 4d [54]. a-tDCS can lead to depolarization of the apical dendritic layer of vertebral neurons (enhanced synaptic input integration) and cell body (Soma) hyperpolarization (reduces action potential output), this “dendritic-cell body decoupling” phenomenon may optimize neural network coding by regulating synaptic weights. For example, changes in dendritic spinous morphology (such as increasing or shrinking volume) can directly affect synaptic transmission efficiency, while gene expression regulation (such as BDNF, Arc genes) further consolidate synaptic plasticity [65]. Anodal stimulation induces depolarization in the apical dendritic layer (blue) of spinal cortical neurons while simultaneously causing hyperpolarization of the soma (red). These changes influence both presynaptic and postsynaptic plasticity, including morphological modifications in dendritic spines, modulation of membrane potentials, alterations in gene expression, regulation of neurotransmitter release, and guidance of axonal development.

The effect of tDCS on non-neuronal cells such as glial and endothelial cells is a relatively new area of research, and some studies have shown that tDCS can affect these cell types, which may have an impact on brain function and vascular health [52]. Fig. 4f.Among the non-neuronal cells in the brain are called neuroglia, which also includes oligodendrocytes, microglia, and astrocytes. They are essential for sustaining the integrity of the cerebral environment, supplying sustenance, assisting in the healing of the nervous system, and controlling neurotransmission. tDCS may enhance its glutamate reuptake ability, reduce excitotoxicity, and indirectly regulate synaptic plasticity by releasing d-serine synergistic NMDAR function [66]. After tDCS stimulation, its activation state may shift from pro-inflammatory (M1 type) to anti-inflammatory (M2 type), reducing neuroinflammatory and promoting damage repair. For example, animal models show that anode tDCS can inhibit IL-1β and TNF-α release while upregulating neurotrophic factors (such as IGF-1) [66]. Meanwhile, tDCS can improve local cerebral blood flow (such as increasing oxygenated hemoglobin concentration) by inducing the release of nitric oxide (NO) and vascular endothelial growth factor (VEGF) [65].

TDCS not only regulates local neuronal activity, but also affects global neural oscillations by changing the functional connection of brain networks. Anodic stimulation can enhance oscillation in the θ (4–8 Hz) and γ (30–100 Hz) bands, promoting working memory and attention; while cathodic stimulation may inhibit β (13–30 Hz) activity and reduce pathological synchronization (e.g. Parkinson's tremor) [63,65]. tDCS targeting DLPFC can enhance the inverse correlation connection between the default mode network (DMN) and the task positive network (TPN), improving emotional regulation and cognitive control in patients with depression [65].

Brain connectivity research primarily focuses on the anatomical pathways, neural connections, and communication mechanisms among various regions of the central nervous system (CNS). A seminal study has provided initial evidence that transcranial direct current stimulation (tDCS) can induce alterations in brain synchronization and modify the topological functional organization of neural networks. The findings suggest that excitatory changes elicited by tDCS may lead to significant reorganization of functional cortical architecture. In this study, four distinct brain networks were simulated during tDCS application, utilizing a matrix-based approach to model connectivity strengths between specific cortical regions. Fig. 4g [67,59].

**Fig. 5:**
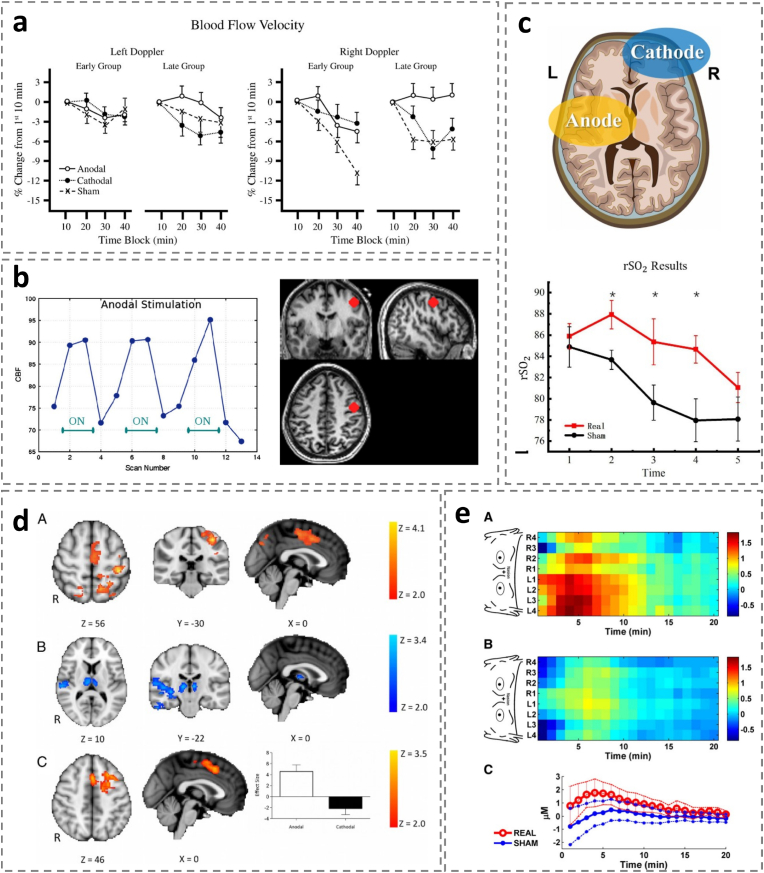
Effects of tDCS on cerebral haemodynamics(*a*)Cerebral oxygenation percent change from baseline. Early Group (stimulation at 10–20 min); Late Group (stimulation at 30–40 min) [74].(b)Changes in rCBF over time in a typical subject fitted with the anodal montage [71].(c)Regional oxygen saturation results [75].(d)Brain perfusion changes during stimulation compared with baseline Red areas represent areas where cerebral perfusion increases during anodic stimulation, and blue areas represent areas where perfusion decreases during cathodic stimulation [69].(e)Spatiotemporal representation of CHbO2 obtained under both real stimulus (top) and sham stimulus (mid) conditions [73].

In addition to EEG and fMRI data, the effects of tDCS on brain connectivity can also be examined based on haemodynamic changes [68,69]. When Nelson et al. explored the role of the dorsolateral prefrontal cortex (DLPFC) in hypervigilance, they showed that anodal and cathodal stimulation resulted in significant increases or decreases in CBFV(cerebral blood flow velocity, cBFV), respectively. Fig. 5a [70]. Zheng et al. measured rCBF (regional cerebral blood flow, rCBF)using magnetic resonance imaging (MRI) and showed that a-tDCS increased rCBF. Fig. 5b [71]. Dalong et al. measured regional cerebral oxygen saturation (rSO_2_) using a wireless cerebral oximetry acquisition system (WORTH headband, Casibrain Technology) across five consecutive time points. Their findings demonstrated a significant increase in rSO_2_ levels within a 4-h observation window. Fig. 5c [72]. Stagg et al. pioneered the combination of left dorsolateral prefrontal cortex (L-DLPFC) stimulation with transcranial direct current stimulation (tDCS) to investigate associated changes in cerebral perfusion pressure (CPP). Their findings revealed distinct patterns of cerebral perfusion: increased perfusion during anodal stimulation (Fig. 5d, top), decreased perfusion during cathodal stimulation (Fig. 5d, middle), and greater perfusion during anodal compared to cathodal stimulation (Fig. 5d, bottom). In a related study, Merzagora et al. utilized functional near-infrared spectroscopy (fNIRS) to monitor changes in oxygenated hemoglobin (HbO2) concentration. They observed that anodal stimulation significantly increased HbO2 levels, with post-stimulation effects persisting for 8–10 min, while cathodal stimulation produced the opposite effect. Fig. 5e [73]. To better quantify these perfusion changes and examine their relationship with cortical states, future research should focus on tDCS-induced modulation of regional oxygen saturation (rSO2) and other cerebral blood flow metrics during task-related activities.

## 3 Transcranial alternating current stimulation

TACS has been successfully used in the modulation of human perception and motor coordination, as well as in the clinical treatment of psychiatric disorders such as Parkinson's disease or schizophrenia.

### 3.1 Parameters of transcranial alternating current stimulation

**Fig. 6:**
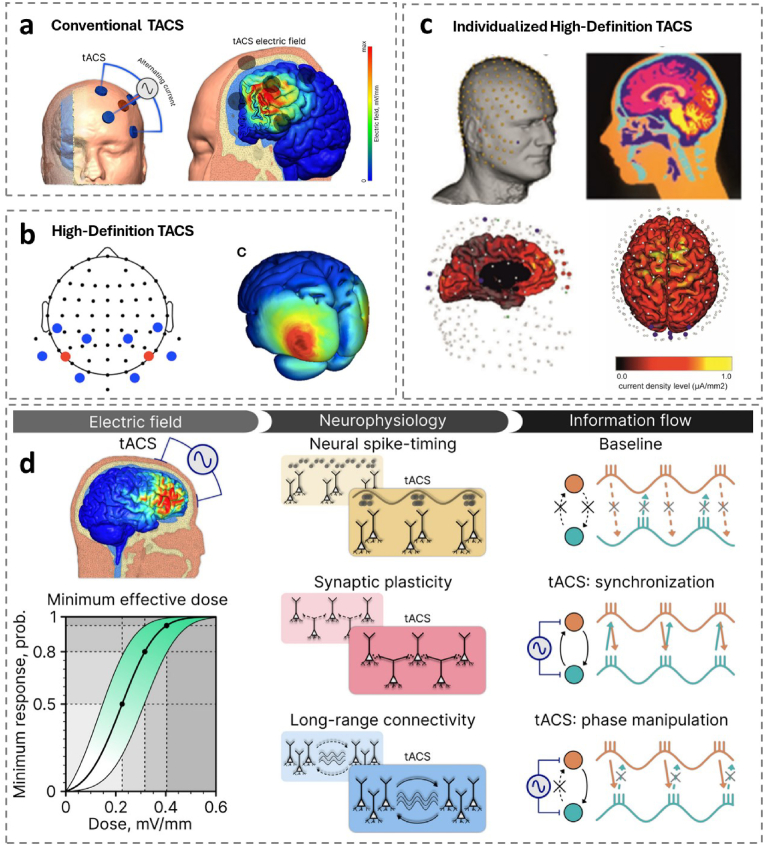
Schematic representation of different types of tACS and their physiological mechanisms.(a)Conventional TACS [80].(b)High-Definition TACS [81].(c)Individualized High-Definition TACS [75].(d)Schematic diagram of the physiological mechanism of tACS [80].

By creating alternating positive and negative voltage changes in particular brain regions, tACS mimics the normal rhythm of electrical activity in the brain [23,26]. This is achieved by using sinusoidal currents. tACS modulates targeted cortical regions through phase-specific voltage oscillations, thereby entraining endogenous brain rhythms to their natural frequency patterns. Fig. 6a [76]. This stimulation is generally performed at low intensity (1–2 mA) for a duration of approximately 10–20 min and is designed to mimic the brain's frequency bands of α、β、γ、δ and θ waves [77,78]. The spatial distribution and magnitude of current flow are determined by electrode placement, individual electrode current intensity (referred to as montage configuration), and regional tissue conductivity profiles. tACS can be administered across a broad frequency spectrum, encompassing conventional electroencephalographic (EEG) ranges (0.1–80 Hz) and extending to higher frequencies (≤140 Hz). [79]. For neuroplasticity investigations, individual sinusoidal waveforms with peak intensities of 0.4–1 mA and frequencies ranging from 10 to 250 Hz have been evaluated. Additionally, higher frequency protocols, extending from near-direct current (DC) levels up to 5 kHz, including single-frequency applications at 200 kHz, have been investigated for their potential in oncological clinical interventions.^32 101^.

Compared to conventional tACS, HD-tACS (high definition transcranial alternating current stimulation, HD-tACS). Fig. 6b [81] and its personalized variants Fig. 6c [81] provide more precise stimulation of brain regions through the use of multiple, small electrodes, thus improving the effectiveness and specificity of stimulation. HD-tACS can be used in unilateral or bilateral configurations and can use different frequencies of stimulation to affect brain function or state, for example using 6 Hz to modulate θ wave activity in the prefrontal cortex thereby affecting the brain's executive function and visuospatial memory [82]. Other frequencies, such as 10 Hz, 20 Hz, and 40 Hz, have also been used to study the effects on hearing [83]、attention [84]、and visuospatial memory [81].

The effect of tACS depends on the intensity of the applied current [80]. The soft tissues surrounding the skull and brain divert approximately 60–75 % of injected current away from the brain, a phenomenon termed the “scalp shunt” effect [85]. Antal et al. note that post-stimulation effects at low intensities (e.g., 0.4 mA) and frequencies (e.g., 1–45 Hz) are negligible due to their minimal magnitude and transient nature. Empirical studies indicate that effective neuromodulation in humans typically requires electric field strengths of 0.3–1 mV/mm, corresponding to total currents of 1–4 mA. Consequently, achieving sufficient electric field intensity is critical for inducing measurable neurophysiological effects [80].

### 3.2 Physiological mechanisms of transcranial alternating current stimulation

Studies on the physiological mechanisms of tACS [86] are summarized below (Table 1):tACS modulates endogenous neural oscillations through precise control of stimulation parameters, including frequency, intensity, and phase alignment. This external rhythmic entrainment promotes neuroplasticity by synchronizing exogenous stimuli with intrinsic brain activity patterns [87,77,88,89].

**Table 1:** Physiological studies related to tACS.

References/Study	Methodology	Targets	Main results
Francis et al. [90]	tACS	Neuronal resonance	tACS can cause cumulative effects across numerous cycles, causing spike timing to shift.
Deans et al. [91]			
Reato et al. [92]			
Kirsch and Nichols [93]	tACS	Cholinergic and adrenergic Neural transmission	The number of presynaptic vesicles decreases and then increases after treatment of reserpine, physostigmine, and tACS.
Zaehle et al. [94]	tACS	Rhythmic patterns and natural pattern	tACS influences neuronal synchronization by increasing or decreasing it, resulting in LTP and LTD.
Fertonani and Miniussi [95]	tACS	–	By promoting or blocking a subthreshold signal, tACS causes stochastic resonance, which influences neuronal groups and causes a wide range of global effects.

Table 1 tACS after-effects on membrane polarity [91,90,92]; tACS after-effects on membrane polarity [93]; tACS after-effects on synaptic plasticity [94]; tACS after-effects on neuronal networks and connectivity [95].

The effects of tACS on brain physiology are realised in three main ways. Firstly, tACS was more effective in modulating the stimulatory effects of concussion in the brain compared to tDCS [96]. tACS induces periodic depolarization and hyperpolarization of neuronal membrane potentials by applying a sinusoidal electric field with frequency specificity (such as α-band 8–12 Hz or γ-band 30–50 Hz). This exogenous rhythm not only resonates with endogenous oscillations (such as α oscillations generated by the thalamic-cortical circuit), but also adjusts synaptic weights through spike time-dependent plasticity [97]. When the tACS frequency matches the inherent oscillation of the target brain region, the spike release time of the neuron population is significantly synchronized, thereby enhancing the power and coherence of the local field potential (LFP) [97]. TACS in different frequency bands have functional heterogeneity in the regulation of neural oscillation. For example, 40 Hz gamma-band stimulation enhances working memory-related frontotopic network synchronization, while the 10 Hz alpha-band improves attention by suppressing the default mode network (DMN) [97]. Some investigations have found that dosages ranging from 0.3 mV/mm to 0.4 mV/mm had an 80 %–95 % likelihood of modulating brain activity. Fig. 6d left [98].

Subsequently, tACS-induced synaptic plasticity depends on the dynamic coupling of NMDA receptor activation to intracellular calcium signaling. When tACS drives periodic fluctuations in neuron membrane potential, postsynaptic depolarization can relieve magnesium ion blockade of NMDA receptors, causing calcium influx to trigger downstream signaling pathways (such as CaMKII, CREB), ultimately promoting long-term enhancement (LTP) [99]. tACS is frequency-dependent on the induction of LTP: low-frequency stimulation (<5 Hz) may trigger long-term inhibition (LTD) through activation of protein phosphatase, while high-frequency stimulation (>10 Hz) preferentially activates the kinase pathway [100]. The tACS-induced LTP enhances signaling between two neurons with effects lasting for hours or even months, which explains the main reason for the long-term offline effect of tACS.Fig. 6d mid [101].

Finally, tACS regulates the functional connection of the distributed brain region through cross-frequency coupling (such as θ-γ oscillation coupling) or homofrequency phase locking. tACS can also be used to modulate phase coherence and long-range connectivity between two or more brain regions. This mechanism enhances information flow within neural networks by precisely synchronizing the timing of neuronal activity, particularly the depolarization threshold required for action potential generation. Through increased coherence, such synchronization promotes more efficient inter-neuronal communication, thereby optimizing the brain's overall communication patterns [102]. As shown, this synchronization may facilitate or inhibit specific types of information flow. Fig. 6d right. This synchronization may promote or suppress a specific type of information flow. For example, applying a stimulus in phase theta band (4–8 Hz) to the prefrontal lobe and hippocampus can enhance phase consistency between the two regions in working memory tasks and improve information transfer efficiency [97].

Most of the stimulation target areas that have been described for TACS to improve muscle strength have focused on the motor cortex [103,104,105]. Its future research direction focuses on how to optimize high-precision electrical stimulation and interference-modulated electrical stimulation, and the optimization schemes are also diverse, such as high-definition TACS, phase-shifted TACS, amplitude-demodulated TACS, time-interference (TI) method, and intersecting short-pulse (ISP) method [106].

## 4 Transcranial random noise stimulation

TRNS is a non-invasive neuromodulation technique that delivers stochastic electrical currents through scalp electrodes to modulate cortical activity. Unlike tACS and tDCS, tRNS employs randomly fluctuating current intensities rather than constant or periodic waveforms. This unique stochastic property has demonstrated therapeutic potential across multiple neurological domains, including visual processing disorders (amblyopia, myopia), cognitive impairments (attention deficits, schizophrenia), language dysfunction, affective disorders, chronic pain management, cerebellar dysfunction, and neurodevelopmental conditions.

### 4.1 Parameters of transcranial random noise stimulation

**Fig. 7:**
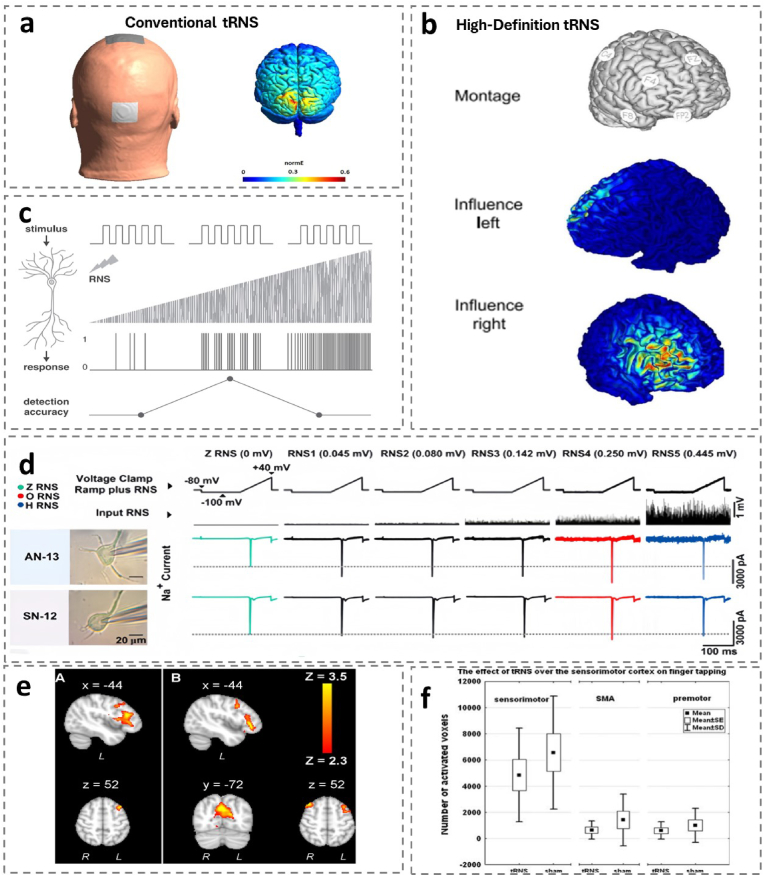
Schematic representation of the different types of tRNS and their physiological mechanisms.(a)Conventional tRNS [110].(b)High-Definition tRNS [109].(c)Conceptual representation of how electrical RNS may enhance the neural signal and influence neural response according to the SR phenomenon [111].(d)The method to analyze the effects of electrical RNS on the peak amplitude of Na + currents elicited by a voltage-clamp-ramp protocol in dissociated cortical neurons of Wistar rats. Left panel, pictures of two pyramidal cells from the auditory and somatosensory cortex. Right panel, voltage-clamp ramps and the associated Na + currents for these cells in conditions of zero RNS and five different levels of RNS as indicated above. Note that there is an increase in the peak amplitude of the Na + current for intermediate intensities of RNS (red recordings) [112].(e)Regions of decreased activity for hf-tRNS. Contrast sham- Hfreq (left) revealed changes in the left frontal cortex. Contrast Lfreq-Hfreq (right) revealed additional changes in right frontal cortex and precuneous [113].(f)Boxplots of the activation volumes resulting from the movement after the diVerent stimulation conditions compared to the REST in the sensorimotor, premotor and SMA [114].

TRNS was formally proposed and widely used in neuroscience in 2008 [107]. As a special form of tACS, the brain is stimulated with alternating current, and the intensity and frequency of the current changes with opportunity, presenting itself as various forms of noise with the characteristics of “white noise”. Fig. 7a [108,107]. For the first time, it was reported by Chenot et al. that HD-tRNS (high-definition tRNS, HD-tRNS) Fig. 7b is more effective than traditional tRNS at enhancing performance in a complex task. The study examined the effects of two different types of tRNS on learning speed, short-term, and long-term performance in a video game [109].

Typically, the tRNS current intensity is between 0.5 and 2 mA, the stimulation duration is 0–20 min, the current density is < 1 A/m^2^, and the current frequency ranges between 0 and 1000 Hz, but lower frequency ranges can be used depending on the target brain area and the desired effect, e.g., low frequency (0.1–100 Hz), high frequency (101–640 Hz), and full frequency (0.1–640 Hz), respectively. Physiological effects on cortical excitability are also different.: high-frequency tRNS (hf-tRNS: 101–640 Hz) increased cortical excitability, whereas low-frequency tRNS (lf-tRNS: 0.1–100 Hz) did not cause significant changes [107].

### 4.2 Physiological mechanisms of transcranial random noise stimulation

One of the core mechanisms of tRNS is to enhance the detection and transmission efficiency of neural signals through random resonance (SR) [111]. SR theory shows that when the system is in a subthreshold state, moderate noise can enhance the detection ability of weak signals. At the neuronal level, tRNS regulates the fluctuations of membrane potential by introducing random electrical noise, bringing it closer to the threshold of action potential [115]. When the noise level is in the optimal range, the discharge probability of the neuron is highly consistent with the timing of the input signal, thereby significantly improving the signal-to-noise ratio (SNR) of the signal. This mechanism is particularly significant in perceptual tasks such as tactile or visual discrimination, and experiments show that tRNS can reduce perceptual threshold by 20 %–30 % and improve the stability of task performance. High-frequency tRNS (hf-tRNS, 100–640 Hz) mainly enhances the activation efficiency of fast sodium ion channels and improves the transient response ability of neurons; while low-frequency tRNS (lf-tRNS, 0.1–100 Hz) adjusts the slow speed Potassium ion channel, affecting the continuous discharge mode of neurons. When a neuron's membrane potential reaches the firing threshold, subthreshold stimuli induce an active depolarization process that generates an all-or-none action potential. Suboptimal noise intensities (too low) prevent weak stimuli from eliciting detectable neural responses. At moderate noise levels, stochastic resonance facilitates precise temporal synchronization between input stimuli and output spikes, thereby optimizing signal detection fidelity. Conversely, excessive noise disrupts this temporal correspondence, degrading detection accuracy through response desynchronization. Fig. 7c.

The enhanced effect of tRNS on cerebral cortex excitability depends mainly on its repeated activation of voltage-gated sodium ion channel (Nav). [116,104,112]. tRNS can increase the probability of opening the Nav channel by depolarizing the film potential, thereby increasing the frequency of distributing the action potential. When analyzing the effect of RNS on the response of Na + current in neurons, it was recorded that under moderate-intensity tRNS, the peak amplitude of sodium current in cells increased significantly (about 30 %–50 %), and this effect was stimulated. It can last for several minutes after the end. (indicated in red records). . tRNS enhances the rapid discharge capability of neurons by regulating the inactivated state recovery rate of the Nav channel. At the same time, tRNS can reduce the activity of GABA neurons and reduce inhibitory postsynaptic currents (IPSCs), thereby improving the excitability of local networks. This mechanism explains the role of tRNS in promoting motor learning ability.

TRNS can not only regulate neuronal activity instantly, but also produce long-term effects by inducing synaptic plasticity. Studies have shown that hf-tRNS can trigger calcium-dependent signaling pathways (such as CaMKII, ERK) by activating NMDA receptors, promoting long-term enhancement (LTP). In addition, tRNS can also enhance synaptic transmission efficiency by regulating the probability of presynaptic vesicles release. Synaptic Structure Remodeling: tRNS increases the stability of postsynaptic dense region (PSD) by regulating the actin dynamics of dendritic spines. Ultra-high resolution imaging showed that the dendritic spine density increased by 15 %–20 % after tRNS stimulation, which was directly related to the enhancement of synaptic plasticity. Meanwhile, tRNS supports the energy demand for synaptic plasticity by enhancing mitochondrial ATP synthesis efficiency. Inhibition of mitochondrial complex I can block the promotion effect of tRNS on LTP, suggesting the key role of energy metabolism in the tRNS effect.

Research using functional magnetic resonance imaging (fMRI) shows that tRNS can induce blood oxygen level-dependent (BOLD) signal changes in the brain, reflecting its far-reaching impact on human cerebral hemodynamics and network function. hf-tRNS is often associated with improvements in early learning ability, manifested as enhanced BOLD signaling in task-related brain regions such as prefrontal and parietal lobes. ^171 178^. lf-tRNS may hinder early learning ability by suppressing the activity of the default mode network (DMN). [113]. Fig. 7e. However, it has also been shown that short-term application of tRNS induces a transient decrease in blood oxygenation level dependent (BOLD) activity in human primary sensorimotor cortex during a finger tapping task [114]. Fig. 7f tRNS can also affect local blood flow by regulating the coupling relationship between neuronal activity and vasodilation. For example, hf-tRNS can increase blood oxygen supply to task-related brain regions, while lf-tRNS may reduce blood flow by inhibiting the expression of vascular endothelial growth factor (VEGF).

TRNS achieves precise regulation of brain function through a multi-scale mechanism, from random resonance enhanced signal detection, sodium ion channel dynamic regulation to blood oxygen network reorganization. Its unique non-invasiveness and frequency specificity provide a new paradigm for the treatment of neuropsychiatric diseases. Future research needs to integrate computational neuroscience, molecular biology and clinical medicine to reveal its whole-brain dynamics laws and promote the implementation of personalized neuroregulatory solutions.

## 5 Safety

Common adverse reactions to the TES technique include a slight tingling sensation, numbness, itchiness, or transient redness of the skin under the electrode plates during stimulation, as well as phosphene, nausea, headache, and dizziness at the onset of stimulation, which are retained for a short period of time, and return to normal after replenishment of saline, rest, and adjustment [117]. For tACS and tRNS, the safe range of current intensity is usually controlled at 1–2 mA (peak-to-peak), and the duration of a single stimulation does not exceed 40 min [118]. It has been shown that tACS maintains safety even with current strengths up to 10 mA (under a specific high-frequency paradigm) when it employs low-frequency (e.g., α、β、θ wave frequencies) or high-frequency (kHz) alternating current (AC) modes [118]. In contrast, random noise current stimulation of tRNS usually uses a current density of 1∼2 mA, and no serious adverse effects have been reported [118].

Using weighted magnetic resonance and 7 T techniques, a number of institutions, including the National Institutes of Health (NIH), the highest level of medical and behavioral research in the United States, and numerous community researchers have scientifically defined the dosimetric safety issue of tDCS. This demonstrates that TES has been tested and found to be safe and effective for humans within reasonable parameters (≤40 min, ≤4 mA, ≤7.2C) [119,120,121,122,123,124]. As with tDCS, the safety of tACS and tRNS as low-intensity TES techniques has been widely validated, with side effects mainly characterized by transient tingling under the electrodes or mild headache and occurring at a lower rate than with tDCS, and no serious adverse events directly related to tACS or tRNS have been identified [2].

Regarding the TES dose, Peterchev and others have scientifically defined the TES dose, which includes the electrode parameters as well as the current stimulation waveforms [125]. For the safety of the entire experimental process, on the one hand, subjects participating in the experiment should be screened for eligibility (e.g., health status, age stage, cognitive level, etc.). Firstly, people who have had an injury or surgery within the last 6 months, who have metal implants in the skull or brain, who suffer from skin disorders, and who are susceptible to seizures, such as epileptic patients with severe medical conditions, should not be subjected to tDCS. In addition, special attention needs to be paid to the match between current frequency and brain waves when using tACS to avoid inducing abnormal neural synchronization activities; tRNS needs to ensure the stability of random noise parameters to prevent discomfort in subjects due to current fluctuations [118].

On the other hand, different experimental phases should be protected accordingly. It is important to choose the right resistor to regulate the current intensity and charge density, especially for tACS and tRNS to ensure that the current density is below the safety threshold of 6.3 A/m^2^ by monitoring the electrode impedance in real time (e.g. with impedance detecting equipment) [118]. It should be noted that the static impedance level of the skin should be within the limits of the tDCS device manufacturer, otherwise it should not be stimulated. The surface of the skin should be wiped with a cotton swab moistened with alcohol before receiving stimulation using round electrodes sufficiently soaked in saline solution. For tACS and tRNS, a pre-stimulation feature (e.g. 0.5 mA pre-adaptive current) is recommended to minimize initial discomfort and to reduce the risk of setup errors with an intuitive operator interface such as a mechanical knob. Stimulation intervals should be reasonably controlled to a sufficiently large extent, primarily to avoid possible skin lesions due to the cumulative effect of the current [126].

## 6 The effect of TES on athletic performance

There is a large body of research data that demonstrates that TES is the key to enhancing human performance and obtaining superior athletic performance, and TES does not fall under the World Anti-Doping Agency (WADA) umbrella, so it can be legally used before and after competition [127,128,129,130,131,132,133]. Much of the research on the link between performance and TES has been done with electrodes placed in the left DLPFC region. The left DLPFC is the main brain region that regulates human sports performance, and its main functions include regulating cognition and emotion, controlling fatigue, enhancing physical recovery, and enhancing motor memory [134]. Stimulation of the DLPFC effectively activates the motor cortex in a state of central nervous fatigue, which determines the organism's ability to continue to perform physical activity effectively [135].

In 2016, Nature reported that the U.S. Ski and Snowboard Association was applying tDCS to elite skiers before races to improve their athletic performance [136]. In 2021, Alexandre Moreira used tDCS on elite U-20 men's and women's football players after matches to accelerate athletes' recovery time after matches [137,138]. In addition to this, Major League Baseball (MLB) [139]、American Hockey [140]、the National Football League (NFL) and the National Basketball Association (NBA) have formally initiated the use of TES equipment for elite athletes or professional sports teams.

Our study did not require further ethics committee approval as it did not involve animal or human clinical trials and was not unethical.

### 6.1 Effects of TES on muscle strength

Muscle strength is defined as the capacity of the neuromuscular system to generate force against external resistance, with neuromodulation and the frequency of neural impulses being critical determinants of muscular performance. Transcranial direct current stimulation (tDCS) demonstrates significant scientific and technological promise for enhancing both muscle strength and endurance. Current research has established that tDCS applied to the dorsolateral prefrontal cortex (DLPFC), primary motor cortex (M1), and temporal cortex effectively enhances upper and lower limb muscle strength in healthy adult populations (Table 2). Notably, Vimolratana et al. demonstrated that a 20-min application of 2 mA anodal tDCS (a-tDCS) over the left M1 region significantly improved dominant limb muscle strength in study participants. [141,142]. Lattari et al. found that 2 mA of a-tDCS intervention on the left motor cortex for 20 min resulted in increased upper limb muscle strength and elevated bench press training volume in subjects [143]. Lerma-Lara et al. found that 2 mA of a-tDCS stimulation of the motor cortex for 20 min resulted in elevated Maximum voluntary contraction (MVC) strength of upper and lower limb muscles in subjects [144]. Hkosaka et al. found that 1.5 mA of a-tDCS intervened on the right motor cortex for 20 min and increased one- and two-handed grip strength in the subjects [145].

**Table 2:** Study Effect of Transcranial Direct Current Stimulation on Muscle strength learning.

Research Literature	Research target	Electrode placement	Electrode specifications/cm^2^	Stimulus duration/min	Stimulus intensity/mA	Campaign programme	Target muscle group	Findings
Behzad Taheri et al., 2024	44 healthy young men	M1	/	20	1.5	80 % of 1 R M perform biceps dumbbell curls	Biceps	tDCS combined with low-intensity exercise with actual blood flow limitation lacks synergistic effects
Elder Nascimento Pereira et al., 2024	80 young and older women	Primary motor cortex	/	30	2	Lung vitality	Respiratory muscles	The strength and lung function of the test group increased.
Zhu et al., 2023	15 healthy young people	a-tDCS: bilateral motor cortex C3 and C4 c-tDCS: ipsilateral over the shoulder	35	20	2	Counter Motion Jumps (CMJs)	muscle strength of the lower limbs	Maximum torque increase at knee, ankle and hip joints
Luo et al., 2023	20 rock climbers	C3,CZ, C4 of bilateral DLPFCs	35	20	2	Level 3 loaded single arm pull down	Latissimus dorsi; obliques	Single Arm Pull Down Explosive Power Lift
Etemadi et al., 2023	14 healthy adult males	a-tDCS: CZ at M1, F3 at left DLPFC c-tDCS: above left shoulder, supraorbital region on AF8	20	20	2	Bicycle ergometry; pedal cadence	Rectus femoris muscle; medial femoris muscle; lateral femoris muscle	Elevated EMG amplitude of the medial femoral muscle
Oranich et al., 2023	18-40 healthy adults	a-tDCS: M1 region at C3 c-tDCS: contralateral orbital region (Fp2)	35	20	2	Supine position; sitting position	Bilateral upper and lower muscles	Advantageous Limb Muscle Strength Enhancement
Lattari et al., 2023	16 healthy adult males	a-tDCS: Left DLPFC	35	20	2	bench press	upper limb muscle	Bench Press Volume Improvement
Xiao, et al., 2022	16 healthy adult males	a-HD-tDCS: C3, C4, FZ, PZ in Cz	3.14	20	2	Short foot exercises; towel curls; toe stretches; squeezes; balance board exercises	flexor digitorum (anatomy)	Foot Movement Function Enhancement
Xiao et al., 2022	30 healthy adults	a-HD-tDCS: C3, C4, FZ, PZ in Cz	3.14	20	2	Ankle Dorsiflexion; Towel Curl; Toe Extension; Squeeze; Balance Board Training	flexor digitorum (anatomy)	Foot Movement Function Enhancement
Ma et al., 2022	12 right-handed male professional rowers	a-tDCS:Left DLPFC	35	20	2	Bilateral knee and shoulder extension	Quadriceps and latissimus dorsi	Left knee and left shoulder isometric muscle lifts
Rodrigues et al., 2022	12 healthy right-handed adult males	Left DLPFC	35	20	2	Bench Press, Back Squat		Back squat load lift
Garcia-sillero et al., 2022	16 male firefighters	a-tDCS: left DLPFC at F3 c-tDCS: frontal cortex above the right eye Fp2	35	20	2	Back squat workout 1 R M (BS exercise)	Rectus femoris, vastus lateralis	Squat exercise speed may increase
Lu et al., 2021	19 healthy adult males	a-tDCS: CZ c-tDCS: at C5 and C6	28	20	2	Maximum casual contraction of the knee joint	Knee extensors and flexors, biceps femoris	Non-dominant leg extensor and flexor MVC lifts
Lerma-Lara et al., 2021	100 healthy adult males	a-tDCS: M1 region	35	20	2	Upper and lower extremity MVC isometric contraction	Biceps brachii, rectus femoris	MVC Strength Improvement for Upper and Lower Body Muscles
Hikosaka et al., 2021	12 healthy adult males	a-tDCS: right M1, C4 area c-tDCS: left M1, C3 zone	25	15	1.5	MVC of grip strength	superficial flexor muscles of the fingers	Increased one-handed and two-handed grip strength

TDCS has been extensively investigated for enhancing lower limb muscle strength in healthy populations. Zhu et al. demonstrated that bilateral application of 2 mA anodal tDCS (a-tDCS) over the primary motor cortex for 20 min significantly enhanced peak torque production across the knee, ankle, and hip joints. [146]. Etemadi et al. found that 2 mA of a-tDCS stimulation of the left motor cortex for 20 min resulted in elevated EMG amplitude of the medial femoral muscles and improved muscle strength of the rectus femoris, medial femoris, and lateral femoris muscles in their subjects [147]. Similarly, Rodrigues et al. found that 2 mA of a-tDCS stimulation of the left motor cortex for 20 min resulted in elevated back squat loads in subjects during bench press and back squat training tests [148]. Xiao et al. conducted a double experiment to address the effect of tDCS on toe flexor strength and found that 2 mA of a-tDCS stimulated the left motor cortex for 20 min, resulting in elevated foot motor function [149,150]. Lu et al. found that 2 mA of a-tDCS intervention on the motor cortex for 20 min elevated MVC in subjects' non-dominant leg extensors and flexors [151].

In addition to studies of ordinary, healthy adult subjects, researchers are also exploring the effects of tDCS on muscle strength in professional athletes and firefighters. lo et al. found that after 20 min of 2 mA a-tDCS stimulation of the bilateral motor cortex of 20 rock climbers, the athletes had elevated one-arm pull-down explosiveness [152]. Ma et al. found that after 20 min of 2 mA a-tDCS stimulation of the left motor cortex in 12 right-handed male professional rowers, the athletes' isometric muscle strength in the left knee and left shoulder was elevated [153]. Garcia-sillero et al. found that after 20 min of 2 mA a-tDCS stimulation of the left motor cortex in 16 male firefighters, the subjects' squatting movement speed may be elevated [154].

However, some of the studies on the effects of tDCS on muscle strength have been obtained inconsistently, suggesting that tDCS does not affect lower limb strength and athletic performance [155]. Jung et al. failed to improve muscular endurance and lower limb explosive strength in 56 healthy adult subjects after a 20-min intervention on the motor cortex using 2 mA of a-tDCS [156]. Savoury et al. intervened in subjects' motor cortex for 10 min using 2 mA of a-tDCS and did not improve subjects' isometric extensor muscle strength [157]. Garner et al. used 2 mA a-tDCS for 20min stimulation of the motor cortex in 18 healthy adults, and the subjects did not improve quadriceps strength [158]. Alibazi et al. tested 12 participants on a task using anodic high-precision tDCS, and found that the muscles were not found to produce maximal grip strength during submaximal grip strength training stimulated with anodic high-precision tDCS, while ipsilateral M1 excitability was not affected [159].

In summary, transcranial electrical stimulation (TES) has demonstrated the capacity to enhance muscle strength performance in subjects when optimal stimulation parameters are applied. Beyond fundamental stimulation parameters, subject-specific factors—including age, health status, and prior professional physical training—significantly influence intervention efficacy. However, the extent to which TES can directly augment muscle strength remains uncertain, as current research lacks large-scale, scientifically rigorous experimental validation in human populations. Furthermore, there is a critical need to integrate neuroimaging techniques to analyze the physiological mechanisms underlying TES-induced muscle strength alterations during intervention periods.

### 6.2 Effect of TES on muscular endurance

The ability of the body's muscles to withstand exhaustion and carry out tasks is known as muscular endurance.

Numerous international studies have investigated the effects of electrical stimulation on the motor cortex in healthy populations. For instance, Etemadi et al. conducted a study involving 14 healthy adult participants, applying 2 mA anodal transcranial direct current stimulation (a-tDCS) to the left motor cortex for 20 min. Their findings revealed that participants receiving tDCS over the dorsolateral prefrontal cortex (DLPFC) exhibited significantly prolonged force maintenance during cycling ergometry compared to control groups. [147]. In a study conducted by Wang et al. a 20-min intervention of anodal transcranial direct current stimulation (a-tDCS) with a current intensity not exceeding 2.2 mA was applied to the motor cortex of 20 healthy, right-handed male college students. The results demonstrated a significant improvement in right elbow flexor endurance among the participants. [160]. Vieira et al. conducted a study in which a 20-min application of 2 mA anodal transcranial direct current stimulation (a-tDCS) was administered to the left motor cortex of 14 healthy adult participants. The results indicated a significant increase in the number of repetitions performed during a moderate-intensity back squat exercise task. [161]. Sidhu et al. conducted a study in which a 10-min application of 2 mA high-definition transcranial direct current stimulation (HD-tDCS) was administered to the left motor cortex of 12 healthy adult participants. The intervention resulted in a measurable improvement in cycling performance among the subjects [162].

Beyond studies involving the general healthy population, numerous researchers have investigated the effects of transcranial electrical stimulation on professional athletes. For instance, Liang et al. applied a 20-min intervention of 2.2 mA anodal transcranial direct current stimulation (a-tDCS) to the motor cortex of eight female rowers. The results demonstrated a 1.05 % improvement in endurance scores during a weight-bearing 5 km rowing task compared to baseline performance. [163]. After a 20-min intervention using 2 mA of a-tDCS on the motor cortex of 13 male basketball players, Chen et al. showed a decrease in fatigue indices after repetitive sprints when the athletes were tested in a 40 × 15 m sprint task [164].

However, conflicting findings have emerged in studies examining the effects of transcranial direct current stimulation (tDCS) on muscle strength. For example, Isis et al. conducted a 20-min intervention using 2 mA anodal tDCS (a-tDCS) on the motor cortex of 15 healthy adult participants. Their results revealed no significant improvement in time to exhaustion (TE) during a maximal incremental cycling exercise test (MIT). [165]. Kristiansen et al. intervened with 13 min of 2 mA electrical stimulation of the motor cortex in 12 healthy subjects and found that the subjects did not improve their performance in a 10 km time trial or cycling [166].

The question of whether tDCS enhances muscular endurance remains an active area of investigation. Some researchers have proposed a novel experimental protocol combining peripheral and central tDCS stimulation. This approach suggests that placing the anodal electrode over the quadriceps motor area and the cathodal electrode over the contralateral supraorbital region may increase peak torque in the quadriceps muscle. [167]. In subsequent explorations, more specific measures should be taken to conduct empirical experiments.

Roberto Monastero et al. In order to evaluate the effects of tRNS applied to cognitive and motor tasks in PD-MCI patients, 10 PD-MCI patients diagnosed according to the Movement Disorder Society Level II MCI criteria received tRNS stimulation. The study showed significant improvements in the patients' motor abilities (Table 3) [168].

**Table 3:** Study Effect of Transcranial Direct Current Stimulation on Muscle muscular endurance.

Research Literature	Research target	Electrode placement	Electrode specifications/cm^2^	Stimulus duration/min	Stimulus intensity/mA	Campaign programme	Target muscle group	Findings
Tai-Chih Chen et al., 2024	20 healthy men	M1 DLPFC	/	20	2	Poppy Jump	lateral femoral muscle	No-Jump Burpee and other physical endurance improvements
Fernanda Ishida Corrêa et al., 2024	32 healthy young women	/	/	20	2	Pelvic Floor Muscle Training (PFMT)	Oral instructions for sitting posture PFMT	tDCS combined with PFMT did not enhance PFMT to increase PFM function in healthy women.
Ângela C Ledur et al., 2024	20 young and healthy women	a-tDCS:M1 c-tDCS:Fp2	/	20	2	Oral instructions for sitting posture PFMT	Oral instructions for sitting posture PFMT	The number of continuous contractions of PFM is improved
Etemadi et al., 2023	14 healthy adult males	a-tDCS: CZ at M1, F3 at left DLPFC c-tDCS: above left shoulder, supraorbital region on AF8	20	20	2	Bicycle ergometry; pedal cadence	Rectus femoris, vastus medialis, vastus lateralis.	The tDCS group of DLPFC had a longer time to exhaustion
Isis et al., 2023	15 healthy adults	a-tDCS; M1/T3 c-tDCS: contralateral supraorbital region	35	20	2	Cycling Maximum Incremental Train (MIT)	Right quadriceps, abdominal muscles	Bike exhaustion time did not improve
Wang et al., 2022	20 healthy right-handed male college students	CZ,F2,C3,C2,C4,PZ	24	20	≤2.2	MVC; maximum elbow flexion exercises	Biceps Triceps	Right elbow flexor endurance improvement
Vieira et al., 2022	11 healthy adult males	a-tDCS: Left DLPFC	35	20	2	Moderate intensity back squat	lower limb muscles	Increased repetitions of the back squat exercise
Liang et al., 2022	8 female rowers	a-tDCS: C2 c-tDCS: C5 and C6	24	20	2.2	Weighted 5 km rowing	lower limb muscles	1.05 per cent improvement in endurance performance from baseline
Chen et al., 2021	13 men's basketball players	a-tDCS: CZ、C5 and C6	24	20	2	40 × 15 m sprint	lower limb muscles	Fatigue index decreased after repeated sprints
Sidhu S K et al., 2021	12 healthy adults	a-tDCS: left motor cortex c-tDCS: right supraorbital region	25	10	2	Cycling programme	Unexercised hand muscles	Decreased short-interval cortical inhibition (SICI) in remote hand muscles after cycling exercise
Kristiansen et al., 2021	12 healthy adults	a-tDCS: DLPFC c-tDCS: DLPFC	25	13	2	10 km cycle time trial	lower limb muscles	Time trial results did not improve

In conclusion, more empirical study is necessary to determine whether the target region for TES placement to improve muscular endurance can be more precisely defined, and some unskilled stimulation regimens should be further validated in the investigation that follows. Future research will investigate the physiological reasons behind the impacts of TES on muscle levels by combining TES technology with other medical sensing technologies, like mirror infrared technology, to synchronize electrical stimulation and brain measurements.

### 6.3 Effect of TES on balance

The human body's most fundamental ability to govern its movement is balance, which can only be improved by postural control system and cognitive function. According to certain research, a-tDCS stimulation of the cerebellum can reduce the body's balance disorders by increasing Purkinje cell activity, enhancing the function of the cerebellar earth region or white matter tracts, and preventing neurons in the deep cerebellar nuclei from producing motor function outputs [169,170].

Balance disorders are among the more common motor dysfunctions, and improvement of balance disorders can be effective in reducing fall-related injuries in older adults. FI Corrêa et al. used a-tDCS to intervene in the motor cortex of healthy older adults, and showed that motor ability, balance, functional independence, and quality of life were improved [171]. Similarly, Glaucio Carneiro Costa et al. used a 2 mA a-tDCS for a 20min intervention on the motor cortex of 28 healthy older adults, and the subjects' balance improved [172]. Songlin Xiao et al. used HD-tDCS to intervene in the motor cortex of 30 and 36 healthy adults, respectively, and the subjects' static balance was significantly improved during balance board training [149,150]. Giancatarina et al. showed an increase in balance in 18 parkour athletes after a 20 min intervention on the motor cortex using 2 mA of tDCS [173].

Balance deficits are also an important cause of sports injuries in athletes. FI Corrêa and Aliasghar Jamebozorgi et al. used a-tDCS to stimulate the primary motor cortex of ACL-injured athletes for 20 min, and after one month, the displacement of the centre of pressure (COP) of the subjects was reduced and the athletes adaptive balance improved [174,175].

However, there are scholars who have come to the opposite conclusion using lower stimulus intensities [176]. Forerster et al. intervened in the right cerebellum of healthy subjects with 1 mA of a-tDCS for 13 min and showed no change in the subjects' static balance (Table 4) [177].

**Table 4:** Study Effect of Transcranial Direct Current Stimulation on balance.

Research Literature	Research target	Electrode placement	Electrode specifications/cm2	Stimulus duration/min	Stimulus intensity/mA	Campaign programme	Evaluation indicators	Findings
Xin Huanget al., 2024	39 healthy young people	Right anode/left cathode cerebellar stimulation, right cathode/left cathode cerebellar stimulation	/	30	2	Stand on the left leg	Swing length, front and rear speed or mid-outside speed	Only after the right cerebellar cathode tDCS is paired with the left cerebellar anode tDCS, can stability be observed
Juho Junget al., 2024	28 middle-aged people	/	/	/	/	Balance training	Swinging when you open your eyes and close your eyes. Functional stretch test and post-intervention investigation	The tDCS group showed significantly greater improvement in static and dynamic balance in terms of sway scores
Raynara Fonseca dos Santos et al., 2024	34 PD patients	a-tDCS: OZ c-tDCS: fp1、fp2	70 × 40 × 18 mm	20	1.5	Balance training	Pose control training	Posture control and balance in Parkinson's disease walkers Improvement
Glaucio Carneiro Costa et al., 2020	28 elderly people	a-tDCS:left DLPFC(F3) c-tDCS: contralateral supraorbital	25、35	20	2	Walk 10 m on a path that contains obstacles. Walk 10 m on carpets of different thicknesses	mini-Balance Evaluation Systems Test (mini-BEST)	Balance Improvement
Aliasghar Jamebozorgi, et al., 2023	33 ACL break athletes	a-tDCS: O1 c-tDCS:O2	25	20	1	10 weeks of intermittent contraction training for lower limb muscles	BEST	Balance Improvement
Songlin Xiao, et al., 2022	30 healthy adults	a-tDCS: Cz c-tDCS: C3, C4, Fz and Pz	3.14	20	2	Foot arches, towel curls, toe stretches and squeezes and balance board exercises	Super Balance	Balance Improvement
Songlin Xiao, et al., 2022	36 participants	a-tDCS:Cz c-tDCS: C3, C4, Fz and Pz	3.14	20	2	Foot Core Exercise (FCE)	Super Balance	Balance Improvement
FI Corrêaet al., 2023	28 elderly people	a-tDCS: left DLPFC c-tDCS: contralateral supraorbital region	35	20	2	30-min walk	BESTest	Improved movement and balance
Giancatarina, et al., 2023	1 8 parkour practice athletes	a-tDCS: FC2	25	20	2	Bipedal and unipedal standing	Centre of Pressure (CoP) Displacement	Balance Improvement

In conclusion, the choice of stimulation program should consider the subject's health status and the various needs for local body posture control when the balance ability of the elderly, neurological patients, or disabled people is impaired. The direction of more in-depth research in the future TES to improve the body's balance is toward the selection of a more optimal, precise, and safe stimulation mode.

### 6.4 Impact of TES on motor skill acquisition

The two main factors impacting the learning of motor skills are human skill practice and central nervous system modulation. The development of creativity and intellectual potential in the human brain has long involved a hemispheric balance hypothesis. However, recent neuroimaging has shown that tDCS stimulation of the primary motor cortex in the left frontal region increases the neural excitability of this cortex and its interconnecting regions, affects NMDAR polymorphisms, and promotes human visual, perceptual, and sensitivity abilities and motor skill learning [178,179,180,181]. However, some researchers have also tested the same protocol on four outstanding deaf 10-m rifle athletes and came to the opposite conclusion [182].

A recent study employed advanced augmented reality (AR) technology integrated with transcranial direct current stimulation (tDCS) to deliver a 20-min intervention targeting the motor cortex of 10 healthy adult participants. The results demonstrated significant improvements in the subjects' folk dance learning and memory capabilities [183]. Similarly, Bisman Mangat et al. used a 1 mA a-tDCS for a 20min intervention on the motor cortex of 36 healthy adult subjects, and the subjects' golf putting learning improved. Anthony W. Meek et al. A 20-min intervention in the motor cortex of 58 healthy adult subjects using 1 mA of a-tDCS resulted in an increase in the subjects' ability to learn to complete a dart-throwing task using their non-dominant hand [184]. In the more complex Finger Tapping Task (FTT) test, Gavin Hsu et al. used a 4 mA a-HD-tDCS for a 12-min intervention on the motor cortex of 108 healthy adult subjects, who showed increased learning ability [185]. In addition to the healthy general population, in a task test for professional athletes, Seung-Bo Park et al. used a 2 mA a-tDCS on 13 professional female volleyball players remembering a 20-min intervention, and the women's volleyball team showed improved dunking speed and consistency [186].

On the other hand, some researchers have reached a different conclusion. Harriet Caesley et al. After a 15-min intervention on the motor cortex of 30 non-dance student participants using 1.5 mA of a-tDCS, the subjects' ability to learn Latin dance did not improve [187]. Laura Flix-Díez et al. After a 20-min intervention in the motor cortex of 23 participants using 1 mA of a-tDCS, subjects' motor learning did not improve [188].

The above findings are closely related to task difficulty [2] 、target site of stimulation [189] and current intensity [190]. Currently, tDCS for motor skill learning is currently developing towards clinical surgical skills ^73 149 180,203 204^and learning field [191] with good results, and the reasonable parameters of the effect of tDCS on motor learning memory in different populations have to be further explored.

In addition to this, TACS and tRNS also affect human motor learning ability to varying degrees [192,193,194,195,196,197]. Samantha J. Bootha et al. showed that the effect of TACS on memory in the human brain was not prominent. TRNS at different frequencies (high frequency, full frequency) affected subjects' motor learning ability differently [198,199]. Stimulus frequency and experimental characteristics are the main factors influencing stimulus effectiveness (Table 5) [199].

**Table 5:** Study Effect of Transcranial Direct Current Stimulation on Motor skill learning.

Research Literature	Research target	Electrode placement	Electrode specifications/cm2	Stimulus duration/min	Stimulus intensity/mA	Campaign programme	Evaluation indicators	Findings
Feng Guo et al., 2024	31 healthy adult men	/	/	20	2	visual isometric pinch task	Overall motor skills learning and speed-accuracy trade-off function	Finger motor skills enhancement
Hakjoo Kimet al., 2024	90 participants	a-tDCS:right M1 c-tDCS:left M1	40 × 40 mm	15	2	serial reaction time task (SRTT)	One training sequence and 5 random sequences	The trajectory of skills development has not changed
Gavin Hsu et al., 2023	108 healthy adults	a-tDCS: above right parietal lobe c-tDCS: above right frontal lobe	2.7	12	4	Finger Tapping Task (FTT)	Number of correct sequences	Finger tapping task learning enhancement
Iris Kico,2022	10 healthy adults	a-tDCS: Cz zone c-tDCS: FPZ zone	20	20	2	Learning steps and learning the whole dance with AR images	Similarities to professional dancers	Dance learning memory enhancement
Bisman Mangat,2022	36 healthy adults	a-tDCS electrode: C1 region c-tDCS: above supraorbital region	25	20	1	75 practice golf putting tasks	Distance of the ball from the centre of the target	Motor learning enhancement
Nirsan Kunaratnam,2022	52 healthy adults	a-tDCS: C3 region of M1 c-tDCS: contralateral supraorbital region	25	20	1	MVC for visuomotor isometric pinch tasks	40 % of the maximum MVC value	Increased ability to acquire motor skills
Iannone et al., 2022	30 right-handed healthy adults	a-tDCS: contralateral M1 of right FDI muscle c-tDCS: ipsilateral supraorbital region	25	20	2	Isometric pinch task approx. 40 min	Whether moving the cursor between “HOME” and the five targets is fast and accurate	Motor skills retention capacity enhancement
Milan Pantovic,2022	4 outstanding deaf 10-m rifle athletes	a-tDCS: on left DLPFC c-tDCS: above contralateral supraorbital region	25	25	2	Daily training tasks for athletes	Distance of the end point of the shot from the centre of the target	Elite deaflympics athletes' rifle shooting scores not improving
Anthony W. Meek,2021	58 young adults	a-tDCS: on M1 on the side opposite the non-dominant hand c-tDCS: on the orbit on the same side as the non-dominant hand	25	20	1	Completion of dart-throwing tasks using the non-dominant hand	Distance between the centre of the bullseye and the tip of the dart	Increased ability to learn in a single training task
Harriet Caesley et al., 2021	30 non-dance student participants	a-tDCS: on C4 (unilateral stimulation) and on C3 and C4 (bilateral stimulation) c-tDCS: lateral part of the corresponding a-tDCS electrode	25	15	1.5	12 Ballroom and Latin Dance Moves	Participants' posture, movement size, timing, arms, legs and overall performance ability	Dance ability hasn't improved
Nam-Gyu Jo et al., 2021	39 healthy adults	a-tDCS: on the motor hotspot of the first dorsal interosseous muscle (FDI) of the non-dominant hemisphere c-tDCS: on the contralateral supraorbital region	35	20	2	Finger Tapping Task (FTT), Grooved Pegboard Test (GPT), and Hand Strength Tests	Hand movement accuracy and reaction time	Finger Tapping Training (FTT)motor skills learning enhancement
Laura Flix-Díez et al., 2021	23 participants	a-tDCS: right (C4) c-tDCS: left (C3) Primary motor cortex (M1)	25	20	1	A 20-min exercise dexterity training programme	Dexterity and sensitivity of hand movements	No impact on motor learning
Seung-Bo Park,2023	13 professional female volleyball players	a-tDCS: CZ zone at the top of the head c-tDCS: C5 and C6	28.16	20	2	Snap performance (snap speed and snap consistency), two vertical jumps (jump and reach: JaR, counter mobile jump: CMJ), bench press and back squat 1 repetition max (1 RM)	Bushnell Velocity Speed Gun Within 16cm2 of the target area.	Increased snap speed and snap consistency

In summary, TES has a positive effect on enhancing human intelligence and creativity. However, the heterogeneity of the TES stimulus parameters, the subjects' ability to recognise and adapt to the task, and the training effect of the subjects all contribute to the differences in the test results.

### 6.5 The effect of TES on athletic recovery

Competitions and stressful events cause transient disturbances in human biopsychological indicators, the main manifestation of which takes the form of increased fatigue in the body. Physical fatigue involves central inhibitions such as increased β power in the brain and synchronization of the bilateral DLPFC [135]. Insufficient rest and recovery time between competitions can be a significant risk factor for injury, and TES technology focuses more on the soothing effect of the central nervous system on the muscles than traditional biofeedback and neuromuscular electrical stimulation, and is more conducive to an athlete's post-competition recovery [200,201].

It has been demonstrated that tDCS can modulate cold, heat and mechanical pain by stimulating the premotor cortex, increasing interhemispheric functional connectivity, and facilitating organismal motor recovery [202,203,204]. Moreira et al. used 2 mA of a-tDCS in two separate 20-min interventions on the left DLPFC of professional football players of different genders in two separate tests. The results of the study showed that the use of tDCS in combination with a recovery training session improved the athletes' perceptual aspects of relaxation and parasympathetic autonomic markers (PAMs) in the post-game, facilitated the recovery of the organism from post-game fatigue, and that the recovery effect exceeded the level of improvement after the recovery training session only (Table 6) [205,206].

**Table 6:** Study Effect of Transcranial Direct Current Stimulation on Sports recovery.

Research Literature	Research target	Electrode placement	Electrode specifications/cm^2^	Stimulus duration/min	Stimulus intensity/mA	Campaign programme	Evaluation indicators	Exercise Recovery effect
Jader Vinicius Da Silva Rocha et al., 2024	27 football players	a-tDCS:M1 c-tDCS:inion	48	15	2	reverse lunge, walking holding the knee unilaterally, isometric squat with arms extended in front, low and medium skipping.	Visual Pain Scale (VAS) and Subjective Recovery Scale (SRS)	The subjective scale did not improve.
Tatlana selitrenikova et al., 2022	20 restlers	a-tDCS:prefrontal cortex c-tDCS:right supraorbital region	35	30	2	Mixed Martial Arts (MMA) sports programme	Heart rate variability (НRV)	Psychophysiological stability and technical and tactical readiness enhancement in MMA athletes
Alexandre Moreira et al., 2021	12 male professional football players	a-tDCS:bilateral DLEFC (F3 and F4) c-tDCS:contralateral supraorbital region	35	20	2	After the official game	Heart rate (HR) Well-Being Questionnaire (WBQ) Sub-polar Running Test (SRT)	Exercise Recovery Enhancement
Jonathan Charest et al., 2021	30 student-athletes	a-tDCS: FPz c-tDCS: Pz	35	20	2	Sleep after exercise	Pittsburgh Sleep Quality Index (PSQI) Epworth Sleepiness Scale (ESS) Michele Sleep Scoring System	PVT mean reaction time are shortened and recovery is enhanced
Mohammad Etoom et al., 2022	84 elite athletes	a-tDCS electrodes: bilateral DLPFC (F3 and F4) c-tDCS; bilateral supraorbital regions FP 1 and FP 2)	1	20	1.5	Sleep after exercise	A-Sleep Monitoring Acti Graph	Increased resilience
Qingchang Wu et al., 2022	90 healthy male students	a-tDCS: central supraorbital region of frontal lobe c-tDCS: vicinity of right and left ear mastoids	45、25	15	1.5	1500 m and 400 m track and field training	Heart rate variability frequency domain index high frequency (HF)	Delayed fatigue and increased recovery

On the other hand, some researchers have reached a different conclusion [207,208]. Fernando et al. concluded that tDCS enhances cycling and running task-enhancing endurance, but has no effect on variables such as HR response, RPE, and exercise-induced muscle pain [209].

## 7 Future perspectives

### 7.1 Further exploration of stimulation parameters and post-induction effects of TES

Recent studies have demonstrated that Transcranial Electrical Stimulation (TES) can significantly enhance brain activity associated with specific neurotransmitter systems and metabolic processes. However, significant heterogeneity has been observed across studies regarding key parameters, including current intensity/density, behavioral paradigms, and participant states (exercise versus rest). Consequently, future research should prioritize the precise optimization of stimulation parameters.

Caution is warranted when applying TES before or during physical exercise, as variations in exercise modalities and stimulation parameters may lead to inconsistent outcomes. Future investigations should focus on elucidating the mechanisms underlying TES-induced effects, particularly its potential for effect regeneration. Additionally, research should emphasize long-term outcomes, including the duration of stimulation effects, their impact on previously unexplored exercise-related outcomes, and potential safety considerations.

### 7.2 Enhanced spatial precision of TES stimulation mode, position, intensity and electrode size

Currently, research on Transcranial Electrical Stimulation (TES) remains in the preliminary exploration stage. Its notable advantages, including painlessness, non-invasiveness, focal precision, and reversibility, offer significant potential for targeted modulation of neural activity in future applications. However, limitations such as the large electrode area persist and require further investigation. In sports disciplines demanding high levels of concentration, such as shooting, or those requiring exceptional spatial perception, such as gymnastics and diving, the effects of TES on athletic performance metrics could be further elucidated through more precise stimulation protocols.

### 7.3 Combined EEG/MEG/fMRI/fNIR to observe the effect of TES on neural activity in the brain

The mechanisms underlying Transcranial Electrical Stimulation (TES) are highly dependent on stimulation parameters, which has led to inconsistent findings across motor performance studies. Additionally, there is limited awareness of its potential effects among researchers and practitioners. To address these challenges, a more systematic approach to controlling TES stimulation parameters and investigating its neurophysiological mechanisms is essential to establish a robust theoretical foundation for enhancing motor performance. For instance, multimodal neurofunctional imaging techniques—such as electroencephalography (EEG), magnetoencephalography (MEG), functional magnetic resonance imaging (fMRI), and functional near-infrared spectroscopy (fNIRS)—could be employed to elucidate these mechanisms. Ultimately, the integration of electrical stimulation with advanced brain measurement technologies, combining electrodes, optical systems, and sensors, holds promise for achieving innovative stimulation outcomes.

### 7.4 Enhancing TES research in the area of sports performance

Transcranial Electrical Stimulation (TES) has been predominantly applied in clinical rehabilitation settings, particularly for psychiatric disorders, brain injury recovery, sensory restoration, emotional regulation, and cognitive enhancement. However, research on its application for performance enhancement in healthy elite athletes remains limited. Notably, professional athletes exhibit distinct physiological characteristics, including differences in cardiovascular, respiratory, and athletic performance metrics, compared to the general healthy population. Future research should focus on optimizing TES intervention parameters, including precise cortical targeting and dosage determination for athletes. Additionally, there is a need to develop a comprehensive stimulation index system tailored to professional athletes and to further validate the efficacy of TES in improving key athletic performance metrics, such as muscle strength, endurance, and balance.

## 8 Summary

Transcranial Electrical Stimulation (TES) represents a significant advancement in neuromodulation technology, primarily employed in clinical rehabilitation for psychiatric disorders, brain injury recovery, sensory restoration, emotional regulation, and cognitive enhancement. The increasing global emphasis on athletic performance enhancement, particularly in the context of international sports competitions such as the Olympic Games, has led to the recent integration of TES technology into sports science applications. Current research identifies three principal TES modalities: transcranial direct current stimulation (tDCS), transcranial alternating current stimulation (tACS), and transcranial random noise stimulation (tRNS). Empirical studies have demonstrated the efficacy of these modalities in enhancing various athletic performance metrics, including muscular strength, endurance, motor skill acquisition, balance control, and post-exercise recovery. TES techniques have been shown to be safe and effective within reasonable parameters (current intensity ≤4 mA and stimulation time ≤40 min). Common adverse effects include mild tingling under the electrodes, transient headache, or skin redness, which are usually relieved by saline supplementation and rest. tACS and tRNS, whose current intensity is usually controlled at 1–2 mA, have been clinically validated for safety, and serious adverse events are rare. To ensure safety, subjects need to be strictly screened (e.g., exclude epileptic patients or those with intracranial metal implants, etc.), and current density and impedance need to be monitored in real time to avoid skin lesions and abnormal neural activity. Overall, the TES technique has a high safety profile under standardized practice.

Given the current challenges and research needs, it is essential to integrate transcranial electrical stimulation (TES) technology with multimodal neurofunctional imaging techniques, such as electroencephalography (EEG), magnetoencephalography (MEG), functional magnetic resonance imaging (fMRI), and functional near-infrared spectroscopy (fNIRS). These techniques enable simultaneous electrical stimulation and real-time monitoring of brain perfusion, combining electrodes, optical systems, and sensors to achieve innovative therapeutic outcomes. Furthermore, the development of flexible, multimodal micro-scale electrical stimulators represents a promising direction to address the limitations of conventional stimulators, including their bulky design and operational complexity.

In summary, TES represents a potent and efficient technology for real-time modulation of brain physiology and motor performance. With advancements in multimodal neuroimaging, materials engineering, and precise stimulation targeting, TES is poised to enter a new era of enhancing human motor capabilities. Looking ahead, this technology holds significant potential for the development of advanced micro-scale implantable electrical stimulation devices in clinical medicine. Such innovations could enable patients with motor disabilities to regain functional mobility and perform essential activities of daily living.

## CRediT authorship contribution statement

**Jingfeng Wang:** Formal analysis, Data curation, Conceptualization. **Li Wu:** Resources, Methodology. **Mingming Sun:** Writing – original draft, Software. **Yuxiang Wu:** Writing – review & editing, Writing – original draft, Visualization, Validation, Supervision, Software, Resources, Project administration, Methodology, Investigation, Funding acquisition, Formal analysis, Data curation, Conceptualization.

## Disclosure statement

No potential conflict of interest was reported by the author(s).

## Declaration of competing interest

The authors declare the following financial interests/personal relationships which may be considered as potential competing interests:Yuxiang Wu reports financial support was provided by Jianghan University. If there are other authors, they declare that they have no known competing financial interests or personal relationships that could have appeared to influence the work reported in this paper.

## Data Availability

No data was used for the research described in the article.

## References

[1] Stagg C.J., Antal A., Nitsche M.A. (2018). Physiology of transcranial direct current stimulation. J. ECT.

[2] Naro A., Bramanti A., Leo A. (2017). Effects of cerebellar transcranial alternating current stimulation on motor cortex excitability and motor function. Brain Struct. Funct..

[3] Priori A. (2003). Brain polarization in humans: a reappraisal of an old tool for prolonged non-invasive modulation of brain excitability. Clin. Neurophysiol..

[4] Wesley J. (1871).

[5] Merton P.A., Morton H.B. (1980). Stimulation of the cerebral cortex in the intact human subject. Nature.

[6] Guleyupoglu B., Schestatsky P., Edwards D., Fregni F., Bikson M. (2013). Classification of methods in transcranial electrical stimulation (tES) and evolving strategy from historical approaches to contemporary innovations. J. Neurosci. Methods.

[7] Bestmann S, Walsh V (2017). Transcranial electrical stimulation[J]. Current Biology.

[8] Bikson M. (2021). History and recent advancements and changes in computational modeling methods for transcranial electrical stimulation. Brain Stimul.

[9] Edwards D.J., Cortes M., Wortman-Jutt S. (2017). Transcranial direct current stimulation and sports performance. Front. Hum. Neurosci..

[10] Fertonani A., Miniussi C. (2016). Transcranial electrical stimulation: what we know and do not know about mechanisms. Neuroscientist.

[11] Hong K.-S., Khan M.N.A., Ghafoor U. (2022). Non-invasive transcranial electrical brain stimulation guided by functional near-infrared spectroscopy for targeted neuromodulation: a review. J. Neural. Eng..

[12] Liu A., Vöröslakos M., Kronberg G. (2018). Immediate neurophysiological effects of transcranial electrical stimulation. Nat. Commun..

[13] Wexler A. (2017). Recurrent themes in the history of the home use of electrical stimulation: transcranial direct current stimulation (tDCS) and the medical battery (1870–1920). Brain Stimul.

[14] Teplan M. (2002). Fundamentals of EEG measurement. Meas. Sci. Rev..

[15] Nitsche M.A., Bikson M. (2017). Extending the parameter range for tDCS: safety and tolerability of 4 mA stimulation. Brain Stimul..

[16] Kronberg G., Bridi M., Abel T., Bikson M., Parra L.C. (2017). Direct current stimulation modulates LTP and LTD: activity dependence and dendritic effects. Brain Stimul..

[17] Bjekić J., Paunovic D., Živanović M. (2022). Determining the individual theta frequency for associative memory targeted personalized transcranial brain stimulation. J. Personalized Med..

[18] Klírová M., Voráčková V., Horáček J. (2021). Modulating inhibitory control processes using individualized high definition theta transcranial alternating current stimulation (HD θ-tACS) of the anterior cingulate and medial prefrontal cortex. Front. Syst. Neurosci..

[19] Batsikadze G., Moliadze V., Paulus W., Kuo M.F., Nitsche M. (2013). Partially non‐linear stimulation intensity‐dependent effects of direct current stimulation on motor cortex excitability in humans. The Journal of physiology.

[20] Frase L., Piosczyk H., Zittel S. (2016). Modulation of total sleep time by transcranial direct current stimulation (tDCS). Neuropsychopharmacology.

[21] Neuling T., Zaehle T., Herrmann C. (2010). Simultaneous recording of EEG and transcranial electric stimulation. Int. J. Psychophysiol..

[22] Agnihotri S.K., Cai J. (2024). Investigating the effects of transcranial alternating current stimulation on cortical oscillations and network dynamics. Brain Sci.

[23] Hannah H.-P., Elizabeth R., Tad T.B. (2024). Effects of transcranial electrical stimulation on physiological responses to acute stress: a systematic review. J. Cogn. Enhanc..

[24] Mahdi Moeini Kouchaksaraei M., Nowshiravan Rahatabad F., Sheikhani A. (2024). Effects of tDCS and tRNS in two electrode placement methods on the excitability of basal ganglia cells in Parkinson’s disease compared to DBS. Biomed. Signal Process Control.

[25] Pushpal D., Carmelo Mario V., Mojtaba S. (2024). Non-invasive brain stimulation in research and therapy. Sci. Rep..

[26] Shuo Q., Lei C., Qingchun W. (2024). The physiological mechanisms of transcranial direct current stimulation to enhance motor performance: a narrative review. Biology (Basel).

[27] Wang Y., Wang J., Zhang Q.-F. (2023). Neural mechanism underlying task-specific enhancement of motor learning by concurrent transcranial direct current stimulation. Neurosci. Bull..

[28] Aloi D, Jalali R, Tilsley P (2022). tDCS modulates effective connectivity during motor command following; a potential therapeutic target for disorders of consciousness[J]. Neuroimage.

[29] Chiang H.-S., Chen M.-Y., Liao L.-S. (2022). Cognitive depression detection cyber-medical system based on EEG analysis and deep learning approaches. IEEE J. Biomedi. Health Inform..

[30] Echevarria M.A.N., Batistuzzo M.C., Silva R.M.F. (2024). Increases in functional connectivity between the default mode network and sensorimotor network correlate with symptomatic improvement after transcranial direct current stimulation for obsessive-compulsive disorder. J. Affect. Disord..

[31] Li M., Cheng D., Chen C., Zhou X. (2023). High-definition transcranial direct current stimulation (HD-tDCS) of the left middle temporal gyrus (LMTG) improves mathematical reasoning. Brain Topogr.

[32] Li W.O., Yu C.K.-C., Yuen K.S.L. (2022). A systematic examination of the neural correlates of subjective time perception with fMRI and tDCS. Neuroimage.

[33] Liebrand M., Karabanov A., Antonenko D. (2020). Beneficial effects of cerebellar tDCS on motor learning are associated with altered putamen-cerebellar connectivity: a simultaneous tDCS-fMRI study. Neuroimage.

[34] McKendrick R., Parasuraman R., Murtza R. (2016). Into the wild: neuroergonomic differentiation of hand-held and augmented reality wearable displays during outdoor navigation with functional near infrared spectroscopy. Front. Hum. Neurosci..

[35] Soleimani G., Towhidkhah F., Oghabian M.A., Ekhtiari H. (2022). DLPFC stimulation alters large-scale brain networks connectivity during a drug cue reactivity task: a tDCS-fMRI study. Front. Syst. Neurosci..

[36] Tu Y, Cao J, Guler S (2021). Perturbing fMRI brain dynamics using transcranial direct current stimulation[J]. NeuroImage.

[37] Bevilacqua M., Feroldi S., Windel F. (2024). Single session cross-frequency bifocal tACS modulates visual motion network activity in young healthy population and stroke patients. Brain Stimul.

[38] Fang Z., Hu D., Zheng R. (2024). Multiple artifact detection based on adaptive scalp region selection and classifier fusion. IEEE Sens. J..

[39] Haslacher D., Nasr K., Robinson S.E., Braun C., Soekadar S.R. (2021). Stimulation artifact source separation (SASS) for assessing electric brain oscillations during transcranial alternating current stimulation (tACS). Neuroimage.

[40] Radecke J.-O., Fiene M., Misselhorn J. (2023). Personalized alpha-tACS targeting left posterior parietal cortex modulates visuo-spatial attention and posterior evoked EEG activity. Brain Stimul.

[41] Rostami M., Zomorrodi R., Rostami R., Hosseinzadeh G.-A. (2022). Impact of methodological variability on EEG responses evoked by transcranial magnetic stimulation: a meta-analysis. Clin. Neurophysiol..

[42] Sehatpour P., Dondé C., Hoptman M.J. (2020). Network-level mechanisms underlying effects of transcranial direct current stimulation (tDCS) on visuomotor learning. Neuroimage.

[43] Chen L.-C., Sandmann P., Thorne J.D., Herrmann C.S., Debener S. (2015). Association of concurrent fNIRS and EEG signatures in response to auditory and visual stimuli. Brain Topogr.

[44] Chiang H.-S., Motes M., O’Hair R. (2021). Baseline delayed verbal recall predicts response to high definition transcranial direct current stimulation targeting the superior medial frontal cortex. Neurosci. Lett..

[45] Figeys M., Zeeman M., Kim E.S. (2021). Effects of transcranial direct current stimulation (tDCS) on cognitive performance and cerebral oxygen hemodynamics: a systematic review. Front. Hum. Neurosci..

[46] Lu H., Wang X., Zhang Y. (2023). Increased interbrain synchronization and neural efficiency of the frontal cortex to enhance human coordinative behavior: a combined hyper-tES and fNIRS study. Neuroimage.

[47] Müller D., Habel U., Brodkin E.S., Clemens B., Weidler C. (2023). HD-tDCS induced changes in resting-state functional connectivity: insights from EF modeling. Brain Stimul.

[48] Wu H., Lu B., Zhang Y., Li T. (2024). Differences in prefrontal cortex activation in Chinese college students with different severities of depressive symptoms: a large sample of functional near-infrared spectroscopy (fNIRS) findings. J. Affect. Disord..

[49] Annavarapu R N, Kathi S, Vadla V K (2019). Non-invasive imaging modalities to study neurodegenerative diseases of aging brain[J]. Journal of chemical neuroanatomy.

[50] Masina F., Montemurro S., Marino M. (2022). State-dependent tDCS modulation of the somatomotor network: a MEG study. Clin. Neurophysiol..

[51] Matsushita R., Puschmann S., Baillet S., Zatorre R.J. (2021). Inhibitory effect of tDCS on auditory evoked response: simultaneous MEG-tDCS reveals causal role of right auditory cortex in pitch learning. Neuroimage.

[52] Morya E., Monte-Silva K., Bikson M. (2019). Beyond the target area: an integrative view of tDCS-induced motor cortex modulation in patients and athletes. J. NeuroEng. Rehabil..

[53] Alam M., Truong D.Q., Khadka N., Bikson M. (2016). Spatial and polarity precision of concentric high-definition transcranial direct current stimulation (HD-tDCS). Phys. Med. Biol..

[54] Graham L.J., Aspart F., Remme M.W.H., Obermayer K. (2018). Differential polarization of cortical pyramidal neuron dendrites through weak extracellular fields. PLoS Comput. Biol..

[55] Giordano J., Bikson M., Kappenman E.S. (2017). Mechanisms and effects of transcranial direct current stimulation. Dose Response.

[56] Cancel L.M., Arias K., Bikson M., Tarbell J.M. (2018). Direct current stimulation of endothelial monolayers induces a transient and reversible increase in transport due to the electroosmotic effect. Sci. Rep..

[57] Monai H., Hirase H. (2018). Astrocytes as a target of transcranial direct current stimulation (tDCS) to treat depression. Neurosci. Res..

[58] Monai H., Ohkura M., Tanaka M. (2016). Calcium imaging reveals glial involvement in transcranial direct current stimulation-induced plasticity in mouse brain. Nat. Commun..

[59] Polanía R., Nitsche M.A., Paulus W. (2011). Modulating functional connectivity patterns and topological functional organization of the human brain with transcranial direct current stimulation. Hum. Brain Mapp..

[60] Liu A., Voroslakos M., Kronberg G. (2018). Immediate neurophysiological effects of transcranial electrical stimulation. Nat. Commun..

[61] Shirehjini S.N., Farahani M.S., Ibrahim M.K. (2023). Mechanisms of action of noninvasive brain stimulation with weak non-constant current stimulation approaches. Iran. J. Psychiatry.

[62] Gartside I.B. (1968). Mechanisms of sustained increases of firing rate of neurones in the rat cerebral cortex after polarization: role of protein synthesis. Nature.

[63] Hill A.T., Rogasch N.C., Fitzgerald P.B., Hoy K.E. (2017). Effects of prefrontal bipolar and high-definition transcranial direct current stimulation on cortical reactivity and working memory in healthy adults. Neuroimage.

[64] Fertonani A., Miniussi C. (2017). Transcranial electrical stimulation: what we know and do not know about mechanisms. Neurosci. A Review J. Bringing Neurobiol. Neurol. amp; Psych..

[65] Woodham R.D., Selvaraj S., Lajmi N. (2024). Home-based transcranial direct current stimulation treatment for major depressive disorder: a fully remote phase 2 randomized sham-controlled trial. Nat. Med..

[66] Donnelly C.R., Andriessen A.S., Chen G. (2020). Central nervous system targets: glial cell mechanisms in chronic pain. Neurotherapeutics.

[67] Cao Jun (2022). Brain functional and effective connectivity based on electroencephalography recordings: a review. Hum. Brain Mapp..

[68] Figeys M., Zeeman M., Kim E.S. (2021). Effects of transcranial direct current stimulation (tDCS) on cognitive performance and cerebral oxygen hemodynamics: a systematic review. Front. Hum. Neurosci..

[69] Stagg C.J., Lin R.L., Mezue M. (2013). Widespread modulation of cerebral perfusion induced during and after transcranial direct current stimulation applied to the left dorsolateral prefrontal cortex. J. Neurosci..

[70] Longden T.A., Hill-Eubanks D.C., Nelson M.T. (2016). Ion channel networks in the control of cerebral blood flow. J. Cerebr. Blood Flow Metabol..

[71] Zheng X., Alsop D.C., Schlaug G. (2011). Effects of transcranial direct current stimulation (tDCS) on human regional cerebral blood flow. Neuroimage.

[72] Dalong G., Yufei Q., Lei Y. (2022). Modulation of thalamic network connectivity using transcranial direct current stimulation based on resting-state functional magnetic resonance imaging to improve hypoxia-induced cognitive impairments. Front. Neurosci..

[73] Merzagora A.C., Foffani G., Panyavin I. (2010). Prefrontal hemodynamic changes produced by anodal direct current stimulation. Neuroimage.

[74] Nelson J.T., McKinley R.A., Golob E.J., Warm J.S., Parasuraman R. (2014). Enhancing vigilance in operators with prefrontal cortex transcranial direct current stimulation (tDCS). Neuroimage.

[75] Dalong G., Yufei Q., Lei Y. (2022). Modulation of thalamic network connectivity using transcranial direct current stimulation based on resting-state functional magnetic resonance imaging to improve hypoxia-induced cognitive impairments. Front. Neurosci..

[76] J. Riddle, F. Frohlich, Targeting neural oscillations with transcranial alternating current stimulation, Brain Res. 2021, 1765: 147491. 10.1016/j.brainres.2021.147491 PMC8206031 33887251

[77] Antal A., Boros K., Poreisz C. (2008). Comparatively weak after-effects of transcranial alternating current stimulation (tACS) on cortical excitability in humans. Brain Stimul..

[78] Moliadze V., Atalay D., Antal A., Paulus W. (2012). Close to threshold transcranial electrical stimulation preferentially activates inhibitory networks before switching to excitation with higher intensities. Brain Stimul..

[79] Moliadze Vera, Andrea (2010). Boosting brain excitability by transcranial high frequency stimulation in the ripple range. J. Physiol. (Paris).

[80] Wischnewski M, Alekseichuk I, Opitz A (2023). Neurocognitive, physiological, and biophysical effects of transcranial alternating current stimulation[J]. Trends in Cognitive Sciences.

[81] Bland N.S., Mattingley J.B., Sale M.V. (2018). No evidence for phase-specific effects of 40 Hz HD–tACS on multiple object tracking. Front. Psychol..

[82] Hosseinian T., Yavari F., Kuo M.-F., Nitsche M.A., Jamil A. (2021). Phase synchronized 6 Hz transcranial electric and magnetic stimulation boosts frontal theta activity and enhances working memory. Neuroimage.

[83] Hyvärinen P., Choi D., Demarchi G., Aarnisalo A.A., Weisz N. (2018). tACS-mediated modulation of the auditory steady-state response as seen with MEG. Hear. Res..

[84] Tesche C, Houck J. 10-Hz tACS up-regulates a visual attention network and down-regulates the rich club[J]. Brain Stimulation: Basic, Translational, and Clinical Research in Neuromodulation, 2023, 16(1): 390.

[85] Mihály V., Takeuchi Y., Brinyiczki K. (2018). Direct effects of transcranial electric stimulation on brain circuits in rats and humans. Nat. Commun..

[86] Korai S.A., Ranieri F., Di Lazzaro V., Papa M., Cirillo G. (2021). Neurobiological after-effects of low intensity transcranial electric stimulation of the human nervous system: from basic mechanisms to metaplasticity. Front. Neurol..

[87] Ali M.M., Sellers K.K., Fröhlich F. (2013). Transcranial alternating current stimulation modulates large-scale cortical network activity by network resonance. J. Neurosci..

[88] Helfrich R.F., Schneider T.R., Rach S. (2014). Entrainment of brain oscillations by transcranial alternating current stimulation. Curr. Biol..

[89] Geraint Rees, Romei (2015). Individual differences in alpha frequency drive crossmodal illusory perception. Current Bio. Cb.

[90] Francis J.T., Gluckman B.J., Schiff S.J. (2003). Sensitivity of neurons to weak electric fields. J. Neurosci..

[91] Deans J.K., Powell A.D., Jefferys J.G. (2007). Sensitivity of coherent oscillations in rat hippocampus to AC electric fields. The Journal of physiology.

[92] Reato D., Rahman A., Bikson M., Parra L.C. (2010). Low-intensity electrical stimulation affects network dynamics by modulating population rate and spike timing. J. Neurosci..

[93] Kirsch D.L., Nichols F. (2013). Cranial electrotherapy stimulation for treatment of anxiety, depression, and insomnia. Psychiatr. Clin..

[94] Zaehle T., Rach S., Herrmann C.S. (2010). Transcranial alternating current stimulation enhances individual alpha activity in human EEG. PLoS One.

[95] Bestmann S., Walsh V. (2017). Transcranial electrical stimulation. Curr. Biol..

[96] Jones K.T., Arciniega H., Berryhill M.E. (2019). Replacing tDCS with theta tACS provides selective, but not general WM benefits. Brain Res..

[97] Elyamany O., Leicht G., Herrmann C.S., Mulert C. (2020). Transcranial alternating current stimulation (tACS): from basic mechanisms towards first applications in psychiatry. Eur. Arch. Psychiatr. Clin. Neurosci..

[98] Alekseichuk I., Wischnewski M., Opitz A. (2022). A minimum effective dose for (transcranial) alternating current stimulation. Brain Stimul..

[99] Ho V.M., Lee J.-A., Martin K.C. (2011). The cell biology of synaptic plasticity. Sci. Technol. Humanit..

[100] Morrison A., Diesmann M., Gerstner W. (2008). Phenomenological models of synaptic plasticity based on spike timing. Biol. Cybern..

[101] Krause M.R., Vieira P.G., Csorba B.A., Pilly P.K., Pack C.C. (2019). Transcranial alternating current stimulation entrains single-neuron activity in the primate brain. Proc. Natl. Acad. Sci..

[102] Fries P. (2015). Rhythms for cognition: communication through coherence. Neuron (Camb., Mass.).

[103] Fröhlich F., Mccormick D.A. (2010). Endogenous electric fields may guide neocortical network activity. Neuron (Camb., Mass.).

[104] Guerra A., Pogosyan A., Nowak M. (2016). Phase dependency of the human primary motor cortex and cholinergic inhibition cancelation during beta tACS. Clin. Neurophysiol..

[105] Sunderam S., Gluckman B., Reato D., Bikson M. (2010). Toward rational design of electrical stimulation strategies for epilepsy control. Epilepsy Behav. B..

[106] Wu L., Liu T., Wang J. (2021). Improving the effect of transcranial alternating current stimulation (tACS): a systematic review. Front. Hum. Neurosci..

[107] Terney D., Chaieb L., Moliadze V., Antal A., Paulus W. (2008). Increasing human brain excitability by transcranial high-frequency random noise stimulation. J. Neurosci..

[108] Antal A., Paulus W. (2013). Transcranial alternating current stimulation (tACS). Front. Hum. Neurosci..

[109] Chenot Q., Hamery C., Lepron E. (2022). Performance after training in a complex cognitive task is enhanced by high-definition transcranial random noise stimulation. Sci. Rep..

[110] Contemori G., Trotter Y., Cottereau B.R., Maniglia M. (2019). tRNS boosts perceptual learning in peripheral vision. Neuropsychologia.

[111] Potok W., van der Groen O., Bächinger M., Edwards D., Wenderoth N. (2022). Transcranial random noise stimulation modulates neural processing of sensory and motor circuits, from potential cellular mechanisms to behavior: a scoping review. eneuro.

[112] Remedios L., Mabil P., Flores-Hernández J. (2019). Effects of short-term random noise electrical stimulation on dissociated pyramidal neurons from the cerebral cortex. Neuroscience.

[113] Avenanti A., Saiote C., Polanía R. (2013). High-frequency TRNS reduces BOLD activity during visuomotor learning. PLoS One.

[114] Chaieb L., Kovacs G., Cziraki C. (2009). Short-duration transcranial random noise stimulation induces blood oxygenation level dependent response attenuation in the human motor cortex. Exp. Brain Res..

[115] Van Der Groen O, Wenderoth N (2016). Transcranial random noise stimulation of visual cortex: stochastic resonance enhances central mechanisms of perception[J]. Journal of Neuroscience.

[116] Chaieb L., Antal A., Paulus W. (2015). Transcranial random noise stimulation-induced plasticity is NMDA-receptor independent but sodium-channel blocker and benzodiazepines sensitive. Front. Neurosci..

[117] Brunoni A.R., Amadera J., Berbel B. (2011). A systematic review on reporting and assessment of adverse effects associated with transcranial direct current stimulation. Int. J. Neuropsychopharmacol..

[118] Antal A., Alekseichuk I., Bikson M. (2017). Low intensity transcranial electric stimulation: safety, ethical, legal regulatory and application guidelines. Clin. Neurophysiol..

[119] Bikson M., Datta A., Elwassif M. (2009). Establishing safety limits for transcranial direct current stimulation. Clinical Neurophysiol. Official J. Int. Federation Clinical Neurophysiol..

[120] Iyer M.B., Mattu U., Grafman J. (2005). Safety and cognitive effect of frontal DC brain polarization in healthy individuals. Neurology.

[121] Iyer P.C., Madhavan S. (2018). Non-invasive brain stimulation in the modulation of cerebral blood flow after stroke: a systematic review of Transcranial Doppler studies. Clin. Neurophysiol..

[122] Stagg C.J., Jayaram G., Pastor D. (2011). Polarity and timing-dependent effects of transcranial direct current stimulation in explicit motor learning. Neuropsychologia.

[123] Gálvez V, Alonzo A, Martin D (2013). Transcranial direct current stimulation treatment protocols: should stimulus intensity be constant or incremental over multiple sessions?[J]. International Journal of Neuropsychopharmacology.

[124] Woods A.J., Antal A., Bikson M. (2016). A technical guide to tDCS, and related non-invasive brain stimulation tools. Clin. Neurophysiol..

[125] Paulus Walter (2013).

[126] Zhao H., Qiao L., Fan D. (2017). Modulation of brain activity with noninvasive transcranial direct current stimulation (tDCS): clinical applications and safety concerns. Front. Psychol..

[127] Amr O., Saba Ş., Hasan K. (2024). Investigations of motor performance with neuromodulation and exoskeleton using leader-follower modality: a tDCS study. Exp. Brain Res..

[128] Fengxue Q., Na Z., Michael A.N. (2025). Effects of dual-site anodal transcranial direct current stimulation on attention, decision-making, and working memory during sports fatigue in elite soccer athletes. J. Integr. Neurosci..

[129] Forouzan F., Mihaly V., Andrew M.B. (2025). Repeated tDCS at clinically-relevant field intensity can boost concurrent motor learning in rats. bioRxiv - Animal Behav.Cogn..

[130] Hope E.G.-M., Alan R.N., Marco M., Jared W.S. (2025). Improving locomotor performance with motor imagery and tDCS in young adults. Sci. Rep..

[131] Meng H., Houston M., Zhang Y., Li S. (2024). Exploring the prospects of transcranial electrical stimulation (tES) as a therapeutic intervention for post-stroke motor recovery: a narrative review. Brain Sci.

[132] Mohamad R., Annemarie L., Ashlyn K.F. (2025). Determining the effects of transcranial alternating current stimulation on corticomotor excitability and motor performance: a sham-controlled comparison of four frequencies. Neuroscience.

[133] Sato T., Katagiri N., Suganuma S. (2024). Simulating tDCS electrode placement to stimulate both M1 and SMA enhances motor performance and modulates cortical excitability depending on current flow direction. Front. Neurosci..

[134] Lattari E., Andrade M.L., Filho A.S. (2016). Can transcranial direct current stimulation improve the resistance strength and decrease the rating perceived scale in recreational weight-training experience?. J. Strength Condit Res..

[135] Tanaka M., Ishii A., Watanabe Y. (2013). Neural mechanism of facilitation system during physical fatigue. PLoS One.

[136] Reardon S. (2016). Performance boost paves way for'brain doping': electrical stimulation seems to boost endurance in preliminary studies. Nature.

[137] Moreira A., Machado D., Moscaleski L. (2021). Effect of tDCS on well-being and autonomic function in professional male players after official soccer matches. Physiol. Behav..

[138] Moreira A., Machado D.G.D.S., Bikson M. (2021). Effect of transcranial direct current stimulation on professional female soccer players' recovery following official matches. Percept. Mot. Skills.

[139] Trothen T.J. (2018). Spirituality, Sport, and Doping: More than Just a Game.

[140] Imperatori L.S., Milbourn L., Garasic M.D. (2018). Would the use of safe, cost-effective tDCS tackle rather than cause unfairness in sports?. J. Cogn. Enhanc..

[141] Hadoush H., Al-Jarrah M., Khalil H., Al-Sharman A., Al-Ghazawi S. (2018). Bilateral anodal transcranial direct current stimulation effect on balance and fearing of fall in patient with Parkinson's disease. NeuroRehabilitation.

[142] Vimolratana O., Lackmy-Vallee A., Aneksan B., Hiengkaew V., Klomjai W. (2023). Non-linear dose response effect of cathodal transcranial direct current stimulation on muscle strength in young healthy adults: a randomized controlled study. BMC Sports Sci. Med. Rehabil..

[143] Lattari E., Vieira L.A.F., Santos L.E.R. (2023). Transcranial direct current stimulation combined with or without caffeine: effects on training volume and pain perception. Res. Q. Exerc. Sport.

[144] Lerma-Lara S., De Cherade Montbron M., Guerin M., Cuenca-Martinez F., La Touche R. (2021). Transcranial direct-current stimulation (tDCS) in the primary motor cortex and its effects on sensorimotor function: a quasi-experimental single-blind sham-controlled trial. Sci. Rep..

[145] Hikosaka M., Aramaki Y. (2021). Effects of bilateral transcranial direct current stimulation on simultaneous bimanual handgrip strength. Front. Hum. Neurosci..

[146] Zhiqiang Z., Wei W., Yunqi T., Yu L. (2023). Effects of bilateral extracephalic transcranial direct current stimulation on lower limb kinetics in countermovement jumps. Int. J. Environ. Res. Publ. Health.

[147] Etemadi M., Amiri E., Tadibi V. (2023). Anodal tDCS over the left DLPFC but not M1 increases muscle activity and improves psychophysiological responses, cognitive function, and endurance performance in normobaric hypoxia: a randomized controlled trial. BMC Neurosci..

[148] Rodrigues G.M., Machado S., Faria Vieira L.A. (2022). Effects of anodal transcranial direct current stimulation on training volume and pleasure responses in the back squat exercise following a bench press. J. Strength Condit Res..

[149] Xiao S., Wang B., Yu C. (2022). Effects of intervention combining transcranial direct current stimulation and foot core exercise on sensorimotor function in foot and static balance. J. NeuroEng. Rehabil..

[150] Xiao S., Wang B., Zhang X., Zhou J., Fu W. (2022). Effects of 4 Weeks of high-definition transcranial direct stimulation and foot core exercise on foot sensorimotor function and postural control. Front. Bioeng. Biotechnol..

[151] Lu P., Hanson N.J., Wen L., Guo F., Tian X. (2021). Transcranial direct current stimulation enhances muscle strength of non-dominant knee in healthy young males. Front. Physiol..

[152] Luo J., Fang C., Huang S. (2023). Effects of single session transcranial direct current stimulation on aerobic performance and one arm pull-down explosive force of professional rock climbers. Front. Physiol..

[153] Ma M., Xu Y., Xiang Z. (2022). Functional whole-brain mechanisms underlying effects of tDCS on athletic performance of male rowing athletes revealed by resting-state fMRI. Front. Psychol..

[154] Garcia-Sillero M., Chulvi-Medrano I., Maroto-Izquierdo S. (2022). Effects of preceding transcranial direct current stimulation on movement velocity and EMG signal during the back squat exercise. J. Clin. Med..

[155] Alix-Fages C., Romero-Arenas S., Calderon-Nadal G. (2022). Transcranial direct current stimulation and repeated sprint ability: No effect on sprint performance or ratings of perceived exertion. Eur. J. Sport Sci..

[156] Jung J., Salazar Fajardo J.C., Kim S. (2024). Effect of tDCS combined with physical training on physical performance in a healthy population. Res. Q. Exerc. Sport.

[157] Savoury R.B., Kibele A., Power K.E. (2023). Reduced isometric knee extensor force following anodal transcranial direct current stimulation of the ipsilateral motor cortex. PLoS One.

[158] Garner C.T., Dykstra R.M., Hanson N.J., Miller M.G. (2021). Transcranial direct current stimulation with the halo sport does not improve performance on a three-minute, high intensity cycling test. Int. J. Exerc Sci..

[159] Alibazi R.J., Frazer A.K., Tallent J. (2021). A single session of submaximal grip strength training with or without high-definition anodal-TDCS produces no cross-education of maximal force. Braz. J. Motor Behav..

[160] Wang L., Wang C., Yang H. (2022). Halo sport transcranial direct current stimulation improved muscular endurance performance and neuromuscular efficiency during an isometric submaximal fatiguing elbow flexion task. Front. Hum. Neurosci..

[161] Vieira L.A.F., Lattari E., de Jesus Abreu M.A. (2022). Transcranial direct current stimulation (tDCS) improves back-squat performance in intermediate resistance-training men. Res. Q. Exerc. Sport.

[162] Sidhu S.K. (2021). Remote muscle priming anodal transcranial direct current stimulation attenuates short interval intracortical inhibition and increases time to task failure of a constant workload cycling exercise. Exp. Brain Res..

[163] Liang Z., Zhou J., Jiao F. (2022). Effect of transcranial direct current stimulation on endurance performance in elite female rowers: a pilot, single-blinded study. Brain Sci..

[164] Chen C.H., Chen Y.C., Jiang R.S. (2021). Transcranial direct current stimulation decreases the decline of speed during repeated sprinting in basketball athletes. Int. J. Environ. Res. Publ. Health.

[165] Isis S., Armele D., Paulo G.L. (2023). The effect of tDCS on improving physical performance and attenuating effort perception during maximal dynamic exercise in non-athletes. Neurosci. Lett..

[166] Antal A., Kristiansen M., Thomsen M.J. (2021). Anodal transcranial direct current stimulation increases corticospinal excitability, while performance is unchanged. PLoS One.

[167] Uehara L, Coelho D B, Leal-Junior E C P (2022). Effects of Transcranial Direct Current Stimulation on Muscle Fatigue in Recreational Runners: Randomized, Sham-Controlled, Triple-Blind, Crossover Study—Protocol Study[J]. American Journal of Physical Medicine & Rehabilitation.

[168] Monastero R., Baschi R., Nicoletti A., Pilati L., Brighina F. (2020). Transcranial random noise stimulation over the primary motor cortex in PD-MCI patients: a crossover, randomized, sham-controlled study. J. Neural Transm..

[169] Leila C., Andrea A., Walter P. (2015). Transcranial random noise stimulation-induced plasticity is NMDA-receptor independent but sodium-channel blocker and benzodiazepines sensitive. Front. Neurosci..

[170] Rezaee Z., Kaura S., Solanki D. (2020). Deep cerebellar transcranial direct current stimulation of the dentate nucleus to facilitate standing balance in chronic stroke survivors—a pilot study. Brain Sci..

[171] Corrêa F.I., Carneiro Costa G., Leite Souza P. (2022). Additive effect of transcranial direct current stimulation (tDCS) in combination with multicomponent training on elderly physical function capacity: a randomized, triple blind, controlled trial. Physiother. Theor. Pract..

[172] Costa G.C., Correa J.C.F., Silva S.M. (2020). Effect of transcranial direct current stimulation and multicomponent training on functional capacity in older adults: protocol for a randomized, controlled, double-blind clinical trial. Trials.

[173] Giancatarina M., Grandperrin Y., Nicolier M. (2024). Acute effect of transcranial direct current stimulation (tDCS) on postural control of trained athletes: a randomized controlled trial. PLoS One.

[174] Jamebozorgi A., Rahimi A., Daryabor A., Kazemi S.M., Jamebozorgi F. (2023). The effects of transcranial direct current stimulation (tDCS) and biofeedback on proprioception and functional balance in athletes with ACL-deficiency. Middle East J. Rehabil. Health Stud..

[175] Tohidirad Z., Ehsani F., Bagheri R., Jaberzadeh S. (2023). Priming effects of anodal transcranial direct current stimulation on the effects of conventional physiotherapy on balance and muscle performance in athletes with anterior cruciate ligament injury. J. Sport Rehabil..

[176] Lee Y.S., Yang H.S., Jeong C.J. (2012). The effects of transcranial direct current stimulation on functional movement performance and balance of the lower extremities. J. Phys. Ther. Sci..

[177] Foerster G., Melo L., Mello M., Castro R., Monte-Silva K. (2017). Cerebellar transcranial direct current stimulation (ctDCS) impairs balance control in healthy individuals. Cerebellum.

[178] Baghande H., Fh J.D. (2022). The effect of sports vision training and transcranial direct-current stimulation on -Short-Term visual memory and spatial recognition memory of volleyball Players1. Motor Behav..

[179] Iannone A., Santiago I., Ajao S.T. (2022). Comparing the effects of focal and conventional tDCS on motor skill learning: a proof of principle study. Neurosci. Res..

[180] Jo N.G., Kim G.W., Won Y.H. (2021). Timing-dependent effects of transcranial direct current stimulation on hand motor function in healthy individuals: a randomized controlled study. Brain Sci..

[181] Kunaratnam N., Saumer T.M., Kuan G. (2022). Transcranial direct current stimulation leads to faster acquisition of motor skills, but effects are not maintained at retention. PLoS One.

[182] Pantovic M., Macak D., Cokorilo N. (2022). The influence of transcranial direct current stimulation on shooting performance in elite deaflympic athletes: a case series. J. Funct. Morphol. Kinesiol..

[183] Kico I., Liarokapis F. (2022). Enhancing the learning process of folk dances using augmented reality and non-invasive brain stimulation, Entertain. Comput.

[184] Meek A.W., Greenwell D., Poston B., Riley Z.A. (2021). Anodal tDCS accelerates on-line learning of dart throwing. Neurosci. Lett..

[185] Hsu G., Shereen A.D., Cohen L.G., Parra L.C. (2023). Robust enhancement of motor sequence learning with 4 mA transcranial electric stimulation. Brain Stimul..

[186] Park S.B., Han D.H., Hong J., Lee J.W. (2023). Transcranial direct current stimulation of motor cortex enhances spike performances of professional female volleyball players. J. Mot. Behav..

[187] Caesley H, Sewell I, Gogineni N (2021). Transcranial direct current stimulation does not improve performance in a whole-body movement task[J]. bioRxiv.

[188] Flix-Diez L., Delicado-Miralles M., Gurdiel-Alvarez F. (2021). Reversed polarity bi-tDCS over M1 during a five days motor task training did not influence motor learning. A triple-blind clinical trial. Brain Sci..

[189] Lefebvre S., Jann K., Schmiesing A. (2019). Differences in high-definition transcranial direct current stimulation over the motor hotspot versus the premotor cortex on motor network excitability. Sci. Rep..

[190] Lerner O., Friedman J., Frenkel-Toledo S. (2021). The effect of high-definition transcranial direct current stimulation intensity on motor performance in healthy adults: a randomized controlled trial. J. NeuroEng. Rehabil..

[191] Toth A.J., Ramsbottom N., Constantin C., Milliet A., Campbell M.J. (2021). The effect of expertise, training and neurostimulation on sensory-motor skill in esports. Comput. Hum. Behav..

[192] Berntsen M.B., Cooper N.R., Hughes G., Romei V. (2019). Prefrontal transcranial alternating current stimulation improves motor sequence reproduction. Behav. Brain Res..

[193] Giustiniani A., Tarantino V., Bonaventura R.E., Smirni D., Oliveri M. (2019). Effects of low-gamma tACS on primary motor cortex in implicit motor learning. Behav. Brain Res..

[194] Matteo Bologna, Andrea (2019). Transcranial alternating current stimulation has frequency-dependent effects on motor learning in healthy humans - ScienceDirect. Neuroscience.

[195] Meier A., Krause V., Pollok B. (2014). Early motor memory consolidation: effects of 10 Hz and 20 Hz transcranial alternating current stimulation (tACS) over the left primary motor cortex (M1). Klin. Neurophysiol..

[196] Sugata H., Yagi K., Yazawa S. (2018). Modulation of motor learning capacity by transcranial alternating current stimulation. Neuroscience.

[197] Vanessa K., Anna M., Lars D., Bettina P. (2016). Beta band transcranial alternating (tACS) and direct current stimulation (tDCS) applied after initial learning facilitate retrieval of a motor sequence. Front. Behav. Neurosci..

[198] Albuquerque L.L.D., Fischer K.M., Pauls A.L. (2019). An acute application of transcranial random noise stimulation does not enhance motor skill acquisition or retention in a golf putting task. Hum. Mov. Sci..

[199] Booth S.J., Taylor J.R., Brown L.J., Pobric G. (2022). The effects of transcranial alternating current stimulation on memory performance in healthy adults: a systematic review. Cortex.

[200] Malone J.K., Blake C., Caulfield B.M. (2014). Neuromuscular electrical stimulation during recovery from exercise: a systematic review. J. Strength Condit Res..

[201] Subhani A.R., Kamel N., Mohamad Saad M.N. (2018). Mitigation of stress: new treatment alternatives. Cogn. Neurodyn.

[202] Barwood M.J., Butterworth J., Goodall S. (2016). The effects of direct current stimulation on exercise performance, pacing and perception in temperate and hot environments. Brain Stimul..

[203] Gabor C., Andrea A., Ferdinand H. (2010). Modulatory effects of transcranial direct current stimulation on laser-evoked potentials. Pain Med..

[204] Unger R.H., Lowe M.J., Beall E.B. (2023). Stimulation of the premotor cortex enhances interhemispheric functional connectivity in association with upper limb motor recovery in moderate-to-severe chronic stroke. Brain Connect..

[205] Moreira A., da Silva Machado D.G., Moscaleski L. (2021). Effect of tDCS on well-being and autonomic function in professional male players after official soccer matches. Physiol. Behav..

[206] Moreira A., Machado DGdS., Bikson M. (2021). Effect of transcranial direct current stimulation on professional female soccer players' recovery following official matches. Percept. Mot. Skills.

[207] Dubois P.E., Ossemann M., De F.K. (2013). Postoperative analgesic effect of transcranial direct current stimulation in lumbar spine surgery: a randomized control trial. Clin. J. Pain.

[208] Jensen M.P., Sherlin L.H., Askew R.L. (2013). Effects of non-pharmacological pain treatments on brain states. Clinical Neurophysiol. Official J. Int. Federation Clinical Neurophysiol..

[209] Fertonani Anna, Miniussi Carlo (2017). Transcranial electrical stimulation: what we know and do not know about mechanisms. Neuroscientist.

